# Testing the genetic predictions of a biogeographical model in a dominant endemic Eastern Pacific coral (*Porites panamensis*) using a genetic seascape approach

**DOI:** 10.1002/ece3.734

**Published:** 2013-09-20

**Authors:** Nancy C Saavedra-Sotelo, Luis E Calderon-Aguilera, Héctor Reyes-Bonilla, David A Paz-García, Ramón A López-Pérez, Amilcar Cupul-Magaña, José A Cruz-Barraza, Axayácatl Rocha-Olivares

**Affiliations:** 1 Laboratorio de Ecología Molecular, Departamento de Oceanografía Biológica, CICESECarretera Ensenada-Tijuana No. 3918, Ensenada, Baja California, 22860, México; 2 Laboratorio de Ecología y Pesquerías de la Zona Costera, Departamento de Ecología Marina, CICESECarretera Ensenada-Tijuana No. 3918, Ensenada, Baja California, 22860, México; 3 Departamento de Biología Marina, Universidad Autónoma de Baja California SurA. P. 19, La Paz, Baja California Sur, 23080, México; 4 Laboratorio de Genética para la Conservación, Centro de Investigaciones Biológicas del NoroesteInstituto Politécnico Nacional No. 195, Col. Playa Palo de Santa Rita Sur, La Paz, Baja California Sur, 23096, México; 5 Laboratorio de Ecosistemas Costeros, Departamento de Hidrobiología, UAM-IztapalapaAv. San Rafael Atlixco No. 186, Col. Vicentina, Distrito Federal, 09340, México; 6 Departamento de Ciencias Biológicas, Centro Universitario de la Costa, Universidad de GuadalajaraAv. Universidad de Guadalajara, No. 203, Puerto Vallarta, Jalisco, 48280, México; 7 Instituto de Ciencias del Mar y Limnología, Universidad Nacional Autónoma de México (Unidad Académica Mazatlán)Avenida Joel Montes Camarena s/n, Mazatlán, Sinaloa, 82040, México

**Keywords:** Ecological niche modeling, genetic diversity, genetic structure, hermatypic coral, MaxEnt, relaxed Abundant Center Hypothesis, seascape genetics

## Abstract

The coral fauna of the Eastern Tropical Pacific (ETP) is depauperate and peripheral; hence, it has drawn attention to the factors allowing its survival. Here, we use a genetic seascape approach and ecological niche modeling to unravel the environmental factors correlating with the genetic variation of *Porites panamensis*, a hermatypic coral endemic to the ETP. Specifically, we test if levels of diversity and connectivity are higher among abundant than among depauperate populations, as expected by a geographically relaxed version of the Abundant Center Hypothesis (rel-ACH). Unlike the original ACH, referring to a geographical center of distribution of maximal abundance, the rel-ACH refers only to a center of maximum abundance, irrespective of its geographic position. The patterns of relative abundance of *P. panamensis* in the Mexican Pacific revealed that northern populations from Baja California represent its center of abundance; and southern depauperate populations along the continental margin are peripheral relative to it. Genetic patterns of diversity and structure of nuclear DNA sequences (ribosomal DNA and a single copy open reading frame) and five alloenzymatic loci partially agreed with rel-ACH predictions. We found higher diversity levels in peninsular populations and significant differentiation between peninsular and continental colonies. In addition, continental populations showed higher levels of differentiation and lower connectivity than peninsular populations in the absence of isolation by distance in each region. Some discrepancies with model expectations may relate to the influence of significant habitat discontinuities in the face of limited dispersal potential. Environmental data analyses and niche modeling allowed us to identify temperature, water clarity, and substrate availability as the main factors correlating with patterns of abundance, genetic diversity, and structure, which may hold the key to the survival of *P. panamensis* in the face of widespread environmental degradation.

## Introduction

Viable populations persist where reproduction and survival balance or exceed mortality and emigration. The highest abundance will generally occur in a region where conditions are close to the physiological optima of the species and biotic interactions allow the highest population growth rates (Hoffmann and Blows 1994). This region, called the center of abundance, is akin to the center of mass of the entire species distribution. As ecological conditions deteriorate away from this center, abundance decreases until a limit is reached where viable populations can no longer persist. In peripheral regions, scarcity of viable habitat and resources limits population size and increases population isolation (Brussard 1984). Peripheral populations often have high turnover rates as their persistence depends on the influx from central areas (Brown 1984; Vucetich and Waite 2003). Brown (1984) proposed a generalized pattern of abundance in which the center of abundance occurs at the geographical center of distribution of a species. This biogeographic pattern, known as the Abundant Center Hypothesis (ACH, Hengeveld and Haeck 1982; Pfenninger et al. 2011; Sagarin and Gaines 2002) or Central[Core]-Marginal[Peripheral] Hypothesis (Hutchison 2003; Gapare et al. 2005; Eckert et al. 2008), has drawn considerable theoretical and empirical attention in ecological and evolutionary research (reviewed by Eckert et al. 2008; Sagarin and Gaines 2002; Sagarin et al. 2006). On the other hand, identifying the geographical center and edges of a distribution may prove challenging for some organisms whose ranges do not conform to regular geometric shapes (Brown et al. 1996). In addition, for organisms with strong latitudinal components, such as the linear distributions of coastal species in the eastern Pacific, the effect of latitudinal gradients may be confounded with those hypothesized by the ACH (Guo 2012). Finally, given the increasing number of species in which the ACH has not been found, the hypothesis has been recently questioned as a general biogeographical pattern, drawing considerable detraction from some ecologists who consider it an over simplified assumption (Sagarin and Gaines 2002; Sagarin et al. 2006).

Here, we posit that the relevant “ecological centricity and marginality” of populations transcends the geographic position and can be referred to the geographic pattern of habitat quality across the ecological land- or seascape (Pfenninger et al. 2011; Wagner et al. 2011). Hence, we explicitly replace the center of geographic distribution by the center of abundance, to test for the genetic consequences of the underlying ecological and microevolutionary forces controlling abundance (e.g., favorable conditions at the center of abundance and unfavorable away from it, high turnover, and lower connectivity at the periphery, among others), even though the center of abundance and the geographical center of the distribution may not coincide (i.e., in the absence of ACH sensu stricto). To prevent unproductive semantic arguments, we define this model allowing for “geographically excentric” centers of abundance, functionally analogous to the abundant geographic center of distribution of the ACH, as the “relaxed ACH (rel-ACH)”, in which periphery is defined relative to the center of abundance, thus the adjectives “central” and “abundant” are used interchangeably. In this model, the potential confounding factor stemming from latitudinal gradients becomes moot, as the driving ecological or evolutionary forces behind the gradients become an integral part of those driving the center of abundance, wherever it may be.

The mechanisms leading to geographically unconstrained centers of abundance provide a valuable null model to address fundamental questions about the pattern and degree of genetic structuring among populations and to understand the processes involved in the evolution of stable geographical limits (Hoffmann and Blows 1994; Eckert et al. 2008). A basic prediction of the rel-ACH is the presence of distinct genetic architectures in abundant and depauperate populations. Because of their demographic surplus (i.e., higher natality than mortality rates), abundant populations are more likely to share high levels of connectivity, whereas those at the periphery, experiencing higher turnover, will be more isolated. This results in a gradient of decreasing genetic diversity away from the center, reaching a minimum in peripheral populations due to increased geographic isolation, genetic drift, and directional selection, which will produce higher levels of genetic differentiation (Hoffmann and Blows 1994; Vucetich and Waite 2003). Natural populations do not always conform to this pattern (reviewed by Eckert et al. 2008; Sagarin and Gaines 2002). For instance, instead of acting as sinks, marginal regions may experience diversification and speciation in the presence of local adaptation favored by low levels of maladaptive gene flow from central populations (Sagarin and Gaines 2002; Vucetich and Waite 2003; Eckert et al. 2008; Budd and Pandolfi 2010). These issues are at the core of the debate regarding whether marginal populations are worthy of conservation efforts (Hunter and Hutchinson 1994; Lesica and Allendorf 1995; Budd and Pandolfi 2010).

Embedded in the question of what shapes the spatial distribution of a species lies the influence of environmental and ecological factors determining its presence or absence in the geographic land- or seascape. Once identified, these factors may correlate with genetic patterns of discontinuity and diversity. These so called land- or seascape genetic analyses provide a mechanistic means to advance our understanding on how the environmental landscape influences the processes and patterns of gene flow and local adaptation (Manel et al. 2003). A powerful approach to identify which environmental factors control the distribution of a species is the ecological niche-modeling (ENM) framework. Several ENM approaches have been developed such as the Genetic Algorithm for Rule-Set Prediction (GARP, Stockwell and Peters 1999) and the Maximum Entropy (MaxEnt, Phillips et al. 2006). These methods aim to derive a predictive model of the conditional probability of finding a species across the geographic space given the range of values of environmental covariates that define its niche. The MaxEnt approach uses sophisticated machine-learning methods and has been increasingly used to identify the factors shaping the distribution of a species and to predict potential distributions given environmental change (Moreno et al. 2011; Kumar 2012; Yang et al. 2013). Thus, the urgency in revisiting fundamental questions about species distributional patterns relates to the need to assess present and future impacts on natural ecosystems (Parmesan et al. 2005). For instance, understanding the demographic and genetic processes controlling the genetic structure of marginal populations is fundamental to address the impacts of global climate and environmental changes, given that these populations are the most vulnerable and most likely to mediate the ensuing range shifts produced by those changes (Parmesan 2006; Sagarin et al. 2006).

Tropical coral reefs have been the focus of conservation efforts for decades as they are the most diverse marine ecosystems and provide goods and services worth billions of dollars yearly (Moberg and Folke 1999). Yet, a significant percentage of reef cover has been lost and more is threatened by significant habitat deterioration and fragmentation at local and global scales (Barber et al. 2001; Knowlton 2001; Selig et al. 2010; Wild et al. 2011). Coral communities in the Eastern Tropical Pacific (ETP) are recognized as the most peripheral and isolated at a global scale, which has drawn attention to the factors allowing their survival in the region (Guzman and Cortés 1993; Cortés 1997; Combosch and Vollmer 2011; Baums et al. 2012). However, genetic patterns of ETP hermatypic coral populations have only recently begun to be understood (Paz-García et al. 2008a, 2012; Combosch and Vollmer 2011; Pinzon and LaJeunesse 2011; Saavedra-Sotelo et al. 2011; Aranceta-Garza et al. 2012; Baums et al. 2012; Pinzon et al. 2012).

**Figure 1:**
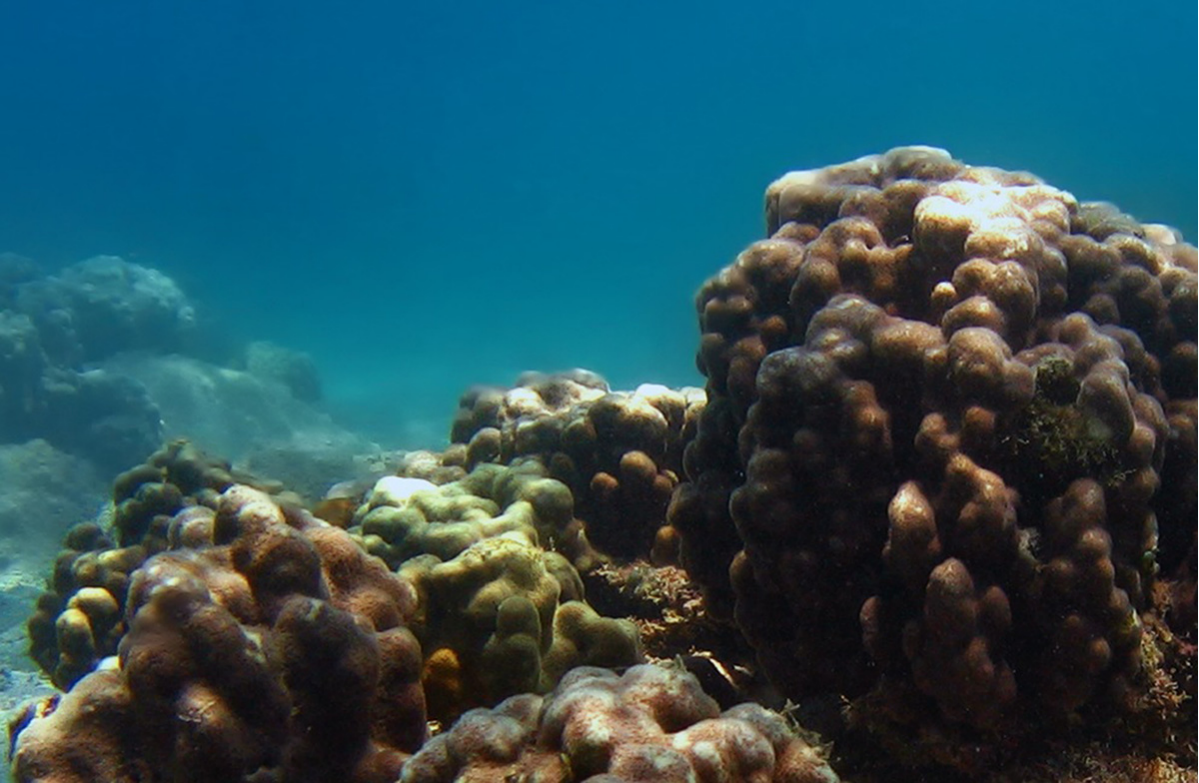
Massive morphotype of the hermatypic coral *Porites panamensis* from La Paz, Baja California Sur, Mexico (Photo: David A. Paz-García).

Here, we address hypotheses regarding the patterns of geographic variation of genetic diversity and structure of a dominant and endemic ETP hermatypic coral in the Mexican Pacific. *Porites panamensis* (Fig. 1) is distributed from the Gulf of California (31°N) to Colombia (3°N) and maybe particularly threatened by global change due to increased vulnerability to warming (Veron 2000). Populations in Central America were severely impacted during the early 80s and late 90s El Niño-Southern Oscillation (ENSO) events (Glynn et al. 1994; Glynn 2000). Sea surface temperature rise as a result of climate change may severely challenge the future of this species, as its reproductive strategy, involving brooding short-lived lecithotrophic planulae that settle near the parental colony, may limit its long-distance dispersal potential (Reyes-Bonilla 1993; Glynn et al. 1994). In light of the above, we use a combination of field surveys, ENM using MaxEnt, and genetic analyses based on nuclear DNA sequences and allozyme electrophoresis, to test the following hypotheses: (1) levels of genetic diversity within populations of *P. panamensis* will correlate with relative abundance, and will decrease away from the center of abundance; (2) populations located near the center of abundance will show higher levels of connectivity and less genetic structure than marginal populations; (3) patterns of diversity and genetic structure predicted by the rel-ACH will be strongly influenced by the presence of significant habitat discontinuities acting as barriers to dispersal; and (4) temperature will be among the most influential environmental variables characterizing the habitat of the species. We found partial agreement with the rel-ACH predictions in the patterns of genetic diversity and structure of colonies of *P. panamensis* sampled from nine populations along the Mexican Pacific. We argue that these patterns are the result of the limited dispersal potential and discontinuity in habitat availability, the latter being strongly influenced by temperature, water clarity, and hard substrate availability.

## Material and Methods

### Patterns of coral abundance

**Figure 2:**
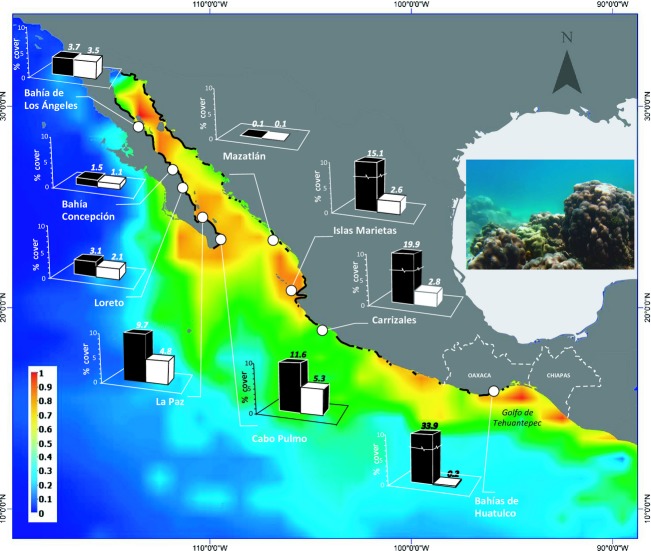
Sampling localities (white circles) and the estimated probability of habitat suitability for *Porites panamensis* using the maximum entropy (MaxEnt) model. Values of estimated probability ranged from 0 (blue color) to 1 (red color). Histograms indicate total live coral cover (black bars) and live coral cover of *P. panamensis* (white bars). Percentages are indicated above each bar; broken black columns represent values out of scale (i.e., >10%). Rocky shorelines are indicated by bold lines.

To assess the geographical patterns of abundance of *P. panamensis* and other zooxanthellate corals in the Mexican Pacific, we conducted surveys between July 2008 and July 2011 at nine localities known to possess live coral colonies of *P. panamensis*: Bahía de los Ángeles (BLA), Bahía Concepción (BC), Loreto (LO), Bahía de La Paz (LP), Cabo Pulmo (CP), Mazatlán (MZ), Islas Marietas (IM), Carrizales (CA), and Bahías de Huatulco (BH) (Fig. 2). Except for Mazatlán, where only one shallow transect was sampled, we surveyed hermatypic coral communities along at least three 25 m-long transects parallel to the coastline at depths 0–6 m, 7–12 m, and >13 m. We estimated percent live coral cover and substratum type in 0.5 × 0.5 m in 10 quadrats placed evenly along transects. Surveying effort was variable and coral cover correlated with effort for all (*P* = 0.004) but not for *P. panamensis* (*P* = 0.926), hence no standardization was necessary for the latter (Table S1; Fig. S1).

### Ecological niche model

To identify the environmental variables that characterize the habitat of *P. panamensis*, we used the MaxEnt approach of ENM to map its probability of potential occurrence in the tropical Mexican Pacific and the Gulf of California (GC). MaxEnt is a machine-learning method particularly suited to model species distributions based on presence-only data (Phillips et al. 2006), which is particularly relevant given the significant challenges in producing reliable “absence” data (see Elith et al. 2011 for a discussion of this issue). The procedure uses environmental data (i.e., covariates) from occurrence records (i.e., presence sites) and from a random sample from the geographic area being modeled (i.e., background landscape data) to estimate the ratio of the conditional probability density of the covariates at the presence sites to the unconditional (i.e., marginal) probability density of the covariates across the studied landscape. The model then estimates the conditional probability that the species is present given the environment (see Dudik et al. 2007 for machine learning and; Elith et al. 2011 for statistical explanations of MaxEnt). Values >0.5 are considered adequate for the establishment of reproductive populations (Franklin 2009; Peterson et al. 2011). We compiled the distributional data of *P. panamensis* from the scientific literature (Reyes-Bonilla et al. 2005) and field surveys from the authors.

Fifteen environmental variables were obtained from public databases at 1° (lat-long) resolution (Table 1). Alkalinity was estimated as a function of sea surface salinity and temperature (Lee et al. 2006); pH and aragonite concentration were estimated using the CO2SYS program (Lewis and Wallace 1998). The model was run with the software MaxEnt v.3.3.3k (Phillips et al. 2004), using a maximum iteration value of 1000, default parameters (convergence threshold of 10^−5^ and automatic regularization of 10^−4^) and logistic output. The geographic output presents the probability of occurrence of the species. The discriminatory power of the model was evaluated on the basis of the area under the curve (AUC) of the threshold independent receiving operating characteristic (ROC) analysis (Phillips et al. 2006), using 75% of the occurrence locations for model training and 25% for testing (Franklin 2009). Jackknife resampling over variables was used to assess their importance for the presence of the species (Elith et al. 2011).

**Table 1:** Environmental variables obtained from public databases used to run Ecological Niche Model with the software MaxEnt v.3.3.3k (Phillips et al., 2004)

Environmental variables	Source Database
Temperature (°C) (yearly mean, maximum and minimum)	MODIS-Aqua satellite available at ftp.cdc.noaa.gov/datasets/noaa.oisst.v2/sst.mmean.mc
Chlorophyll-*a* concentration (mg/m^3^)	
Yearly average of Photosynthetically Active Radiation (PAR, Einstein/m^2^/d)	
Light attenuation coefficient (1/m)	
Nutrient concentrations (Phosphate, Silicate and Nitrates; mM),	World Ocean Atlas available at http://www.nodc.noaa.gov
Dissolved oxygen (mL/L)	
Salinity (PSU)	
Bathymetry (m)	General Bathymetric Chart of the Oceans available at http://www.gebco.net
Atmospheric CO_2_	Scripps Institution of Oceanography, La Jolla, CA (http://scrippsco2.ucsd.edu/data/ljo.html)
Alkalinity estimated as a function of sea surface salinity and temperature	Equations described by Lee et al., 2006
pH and aragonite concentration were estimated from salinity, temperature, alkalinity, silicate, phosphate, and atmospheric CO_2_	Estimates using the CO2SYS program (Lewis & Wallace 1998)

### Tissue sampling and genetic analyses

Tissue samples (5 × 5 cm) were collected from live colonies from August 2008 to November 2011 (Table S1) at the surveyed localities, and included an additional small sample (*n* = 2) from Isla María Madre (Islas Marías, Nayarit). Sampled colonies were separated by at least 2–3 m to avoid inadvertently resampling the same clone. Coral fragments were suspended in preservation buffer (20% dimethyl sulfoxide, saturated NaCl, ethylenediaminetetraacetic acid 250 mmol/L pH 8) (Seutin et al. 1991). DNA was extracted using standard proteinase K digestion and purified with a salting-out protocol, followed by organic extraction, and subsequent ethanol precipitation (Aljanabi and Martínez 1997).

In order to integrate all genetic data available on this species from the Mexican Pacific consisting of multiple genetic markers with different mutation rates, we amplified and sequenced two nuclear DNA loci and reanalyzed five alloenzymatic loci. The ITS1-5.8S-ITS2 region of nuclear ribosomal DNA (rDNA) and an anonymous single copy Open Reading Frame (ORF) were PCR amplified using primers ITS4 and ITS5 (White et al. 1990) and MM32f2 and MM32r2 (C. Prada and M. E. Hellberg, pers. comm.), respectively. The rDNA was analyzed in all available samples from each locality and the ORF in 50% of the organisms (*n* = 12 per locality), owing to its lower level of polymorphism (see Table S2 for PCR conditions and thermal cycling profiles). We reanalyzed data from five alloenzymatic loci (*ME*-1, *GDH*-1, *GDH*-2, *EST*-1, and *LGG*-1) from a previous study of *P. panamensis* in this region. These samples were collected from August 2004 to December 2006 from five localities sampled in this study (Bahía de Los Angeles, Bahía Concepción, La Paz, Islas Marietas–Isla Redonda–, and Bahías de Huatulco –Playa La Entrega–), and an additional site in the southern GC (Punta Arenas –PA–). See Paz-García et al. (2012) for methodological details.

### Genetic and environmental data analyses

DNA sequences were aligned using MEGA v.5 (Tamura et al. 2011) and individual haplotypes identified using DnaSP v.4.0 (Rozas et al. 2003). In organisms showing intragenomic polymorphisms, haplotype reconstruction was conducted by careful examination of chromatograms and with the help of algorithms available in PHASE (Stephens et al. 2001; Stephens and Donnelly 2003), fastPHASE (Scheet and Stephens 2006), and HAPAR (Wang and Xu 2003) (Figs. S2 and S3; Table S3).

To examine the geographic patterns of genetic diversity, we estimated haplotype (*h*) and nucleotide (*π*) diversities using Arlequin v.3.1 (Excoffier et al. 2005). Given the heterogeneity in sample sizes, diversity was standardized to a common sample size using resampling procedures. Haplotype richness (*A*) and the number of private haplotypes (p*A*) were standardized to *n* = 12 and *n* = 21 using the program ADZE v. 1.0 (Szpiech et al. 2008); and haplotype diversity (*h*) to *n* = 12 using a manual resampling procedure in combination with Arlequin v.3.1 (Excoffier et al. 2005). To test patterns of genetic heterogeneity, we used hierarchical Analyses of Variance (ANOVA, *F*-statistics) and of Molecular Variance (AMOVA, *Φ-*statistics) as implemented in Arlequin v.3.1 (Excoffier et al. 2005). For the latter we used a matrix of interhaplotypic distances based on the optimal model of nucleotide evolution (JC+I+G both loci) obtained with MODELTEST v.3.7. (Posada and Crandall 1998). With the aim of testing for barriers to dispersal between central and peripheral populations, we estimated levels of genetic differentiation among localities (*Φ*_st_), between central/peninsular (BLA, BC, LO, LP, PA, and CP: peninsular henceforth) and continental/peripheral (MZ, IM, CA, and BH: continental henceforth) regions (*Φ*_ct_), and among localities within regions (*Φ*_sc_). In addition, we tested for differences between pairs of localities. ANOVA and AMOVA significance was evaluated through permutation tests and adjusted for multiple testing using the sequential Bonferroni correction (Rice 1989).

One of the basic genetic predictions of the rel-ACH is that the central populations are more likely to share high levels of connectivity than peripheral populations; hence, we estimated mutation-scaled levels of effective population size (θ = 2*Neμ*) and gene flow (*M = m/μ)* using a model-based approach involving maximum likelihood Markov Chain Monte Carlo (MCMC), as implemented in MIGRATE v.2.1.3 (Beerli 2004). MIGRATE uses a coalescence-based approach to estimate θ and *M* (magnitude and direction) among populations, assuming a constant per-locus mutation rate (*μ*). We used 10 short-chain searches (5000 genealogies) and three long-chain searches (50,000 genealogies) and a burn-in of 10,000 trees to ensure independence from initial conditions.

To address the influence of habitat characteristics on the genetic patterns of the species, we tested for correlations between genetic diversity and differentiation with environmental covariates. For this analysis, we used the fifteen environmental variables mentioned above and four additional: dissolved inorganic carbon (mol/m^3^), euphotic zone depth (m) (source: MODIS-Aqua satellite, website cited above), total particulate organic carbon (mol/kg) (source: World Ocean Atlas, website cited above), and primary productivity (gC/m^2^/year) (source: http://www.science.oregonstate.edu/ocean.productivity/). First, we reduced the dimensionality of the 19 variables into a new set that accounted for most of the observed variance with a principal component analysis (PCA) of their standardized (mean = 0, SD = 1) values. These principal components (PC) were then correlated with levels of sample size-standardized genetic diversity from each location. As environmental gradients from central to peripheral populations may generate a stepping stone pattern of dispersal in species with limited dispersal capability, we tested the existence of isolation by distance (IBD). In addition, we assessed whether genetic differentiation between pairs of sites correlated with environmental distance, and if the latter correlated with geographic distance, as expected in the presence of an environmental gradient. For this, we constructed matrices of geographical (km), environmental (Euclidean distances from the 19 standardized variables), and genetic (pairwise molecular *Φ*_st_) distances between pairs of sites and performed all pairwise correlations, uncorrected and corrected for the third matrix, with partial Mantel test as implemented in IBD v.1.52 (Bohonak 2002).

The two sequenced (rDNA and ORF) and the five allozyme loci (*ME*-1, *GDH*-1, *GDH*-2, *EST*-1, and *LGG*-1) were used in parallel analyses to test the same fundamental hypotheses regarding the patterns of genetic diversity and structure predicted by the rel-ACH.

## Results

### Patterns of coral abundance in the Mexican Pacific and ecological niche model

The general pattern of abundance of hermatypic corals in the Mexican Pacific features higher coral cover in southern localities decreasing northward with latitude (Fig. 2 black bars). In contrast, the abundance of *P. panamensis* is higher in northern (peninsular) than in southern (continental) localities (Fig. 2 white bars). The highest cover of this species was found in CP (5.3%), LP (4.8%), and BLA (3.5%), all in Baja California; whereas the lowest were found in the continent (BH = 0.25% and MZ = 0.1%). To test for a latitudinal gradient in abundance, we correlated the percent of live coral cover of *P. panamensis*, a measure of absolute abundance, and the ratio of live coral cover of *P. panamensis* to total cover, a measure of relative abundance, with latitude. Even though the trend of *P. panamensis* cover was to increase with latitude, it was not significant (*r* = 0.30 *P* = 0.43, Fig. S4A). In contrast, the relative abundance of *P. panamensis* showed a significant latitudinal gradient (*r* = 0.84 *P* = 0.004; Fig. S4B). This is consistent with the covariance of *P. panamensis* abundance with its level of dominance in coral communities. In northern localities, this species was the dominant zooxanthellate coral contributing with over 68% of total cover in BLA, BC, and LO. In LP and CP, the dominance was less dramatic but the species contributed nearly 50% of total cover. In contrast, the contribution of *P. panamensis* in continental localities was considerably smaller and declined toward the south (IM 17%, CA 14%, and BH 0.6%). These patterns reveal that in the Mexican Pacific, northern peninsular populations represent the center of abundance of the species whereas southern continental localities harbor peripheral depauperate populations. Admittedly, our sampling does not cover the entire range of the species, which extends south to Colombia. However, we expect this pattern of southern depauperate populations to hold based on published abundance patterns (Glynn et al. 1994; Glynn 2000).

The ecological niche model of *P. panamensis* was very robust (AUC = 0.969) and four environmental variables explained ∼77% of the variance: PAR (35.7%), nitrate concentration (16.5%), minimum temperature (15.4%), and bathymetry (9.6%); the remaining were of lesser importance. The results showed the most likely habitat for the species is found in the GC and a few hotspots along the continental Mexican Pacific, such as in the islands close to the entrance of the GC and in the Gulf of Tehuantepec (Fig. 2). Inside the GC, the model maps the presence of suitable rocky habitat on both coasts fairly well (Fig. 2). Even though the probability of occurrence was not correlated with the abundance of *P. panamensis*, as measured by percent live coral cover (*P* = 0.13) or relative abundance (*P* = 0.53), there were some notable parallels. For instance, BLA with the highest probability of occurrence (0.849) had one of the highest coral covers and MZ with a smaller probability of occurrence (0.544) showed consistently the lowest coral cover (Table 2). Among peninsular localities, the sites with a lower coral cover agree with the lowest probability of occurrence (BC 0.698 and LO 0.583). In contrast, the high probability of occurrence (0.704) and the low coral cover observed in BH were contradictory (Table 2).

**Table 2:** Sample size (*n*), number of haplotypes or alleles (*A*), private haplotypes or alleles (p*A*), haplotype diversity (*h* ± SD), nucleotide diversity (π ± SD), observed heterozygosity (*Ho*), expected heterozygosity (*He*), *Porites panamensis* percent live coral cover (% LCC), and MaxEnt derived probability of occurrence (PO)

		rDNA (ITS)											
				
Region	Localities	*n*	*A*	p*A*	*A*_(12)_	p*A*_(12)_	*A*_(21)_	p*A*_(21)_	*h*	*h*_(12)_	*π* (%)	% LCC (±SD)	PO
Peninsula	BLA	29	6	3	3.90	1.51	5.10	2.38	0.63 ± 0.07	0.65 ± 0.11	0.28 ± 0.19	3.50 (±0.81)	0.849
	BC	24	3	0	2.71	0.19	2.99	0.02	0.42 ± 0.11	0.44 ± 0.14	0.08 ± 0.08	1.10 (±0.03)	0.698
	LO	21	6	2	4.65	1.47	6	2.08	0.75 ± 0.06	0.75 ± 0.09	0.45 ± 0.28	2.10 (±0.62)	0.583
	LP	28	9	5	5.48	2.55	7.67	3.99	0.70 ± 0.09	0.71 ± 0.13	0.25 ± 0.18	4.80 (±2.58)	0.746
	CP	30	11	5	6.94	2.74	9.31	4.01	0.88 ± 0.04	0.87 ± 0.07	0.41 ± 0.25	5.30 (±3.96)	0.716
Continent	MZ	21	3	2	2.14	1.14	3	2	0.19 ± 0.11	0.20 ± 0.13	0.03 ± 0.05	0.10 (±0.00)	0.544
	IM	34	10	7	5.52	3.84	7.75	6.06	0.71 ± 0.08	0.70 ± 0.12	0.41 ± 0.26	2.62 (±1.63)	0.745
	CA	12	2	0	2	1.65	n.d.	n.d.	0.17 ± 0.13	0.17 ± 0.13	0.12 ± 0.11	2.83 (±1.56)	0.733
	BH	14	3	2	2.97	1.99	n.d.	n.d.	0.48 ± 0.14	0.48 ± 0.15	0.27 ± 0.19	0.25 (±0.33)	0.704

		ORF					Allozymes (five enzyme systems)				
			
Region	Localities	*n*	*A*	p*A*	*h*	*π* (%)	*n*	*A*	*pA*	*Ho*	*He*
Peninsula	BLA	12	5	2	0.73 ± 0.11	0.35 ± 0.26	20	14	1	0.260	0.583
	BC	12	2	0	0.17 ± 0.13	0.09 ± 0.11	20	13	0	0.180	0.564
	LO	12	3	0	0.62 ± 0.09	0.30 ± 0.23	–	–	–	–	–
	LP	12	3	1	0.44 ± 0.16	0.13 ± 0.14	20	13	0	0.190	0.553
	PA	–	–	–	–	–	34	13	0	0.106	0.479
	CP	12	7	4	0.86 ± 0.08	0.52 ± 0.36	–	–	–	–	–
Continent	MZ	11	4	2	0.69 ± 0.13	0.31 ± 0.25	–	–	–	–	–
	IM	14	2	0	0.14 ± 0.12	0.04 ± 0.07	20	12	0	0.141	0.430
	CA	12	3	1	0.32 ± 0.16	0.09 ± 0.11	–	–	–	–	–
	BH	11	1	0	0	0	25	13	1	0.160	0.533

Locations are categorized as peninsular (BLA, BC, LO, LP, PA, CP) and continental (MZ, IM, CA, BH). Allozyme data are from Paz-García et al. (2012). Subscript in brackets indicates standardized values at minimum sample size (*n* = 12 and *n* = 21). n.d., Not defined and –, no data.

### Intraindividual rDNA polymorphisms

A few (15.3%) rDNA sequences revealed the presence of intragenomic polymorphisms: all instances involved the combination of only two sequence variants. Individual haplotypes from these organisms were completely resolved (Figs. S2 and S3; Table S3). To test the influence of intragenomic polymorphisms on molecular diversity estimates, we compared genetic diversity using two coding strategies. In the first, individuals were considered diploid (i.e., homoplasmic colonies were coded as homozygous and heteroplasmic as heterozygous); in the second, homoplasmic colonies were treated as haploid and heteroplasmic were treated as two haploid individuals, hence, effectively increasing sample sizes in the affected populations (Table S3). We found no significant differences in estimates of *h* between approaches (*t*_480(0.05)_ = 1.17, *P* = 0.24), hence we opted to treat heteroplasmic colonies as independent haploid organisms.

### Geographic patterns of genetic diversity

**Figure 3:**
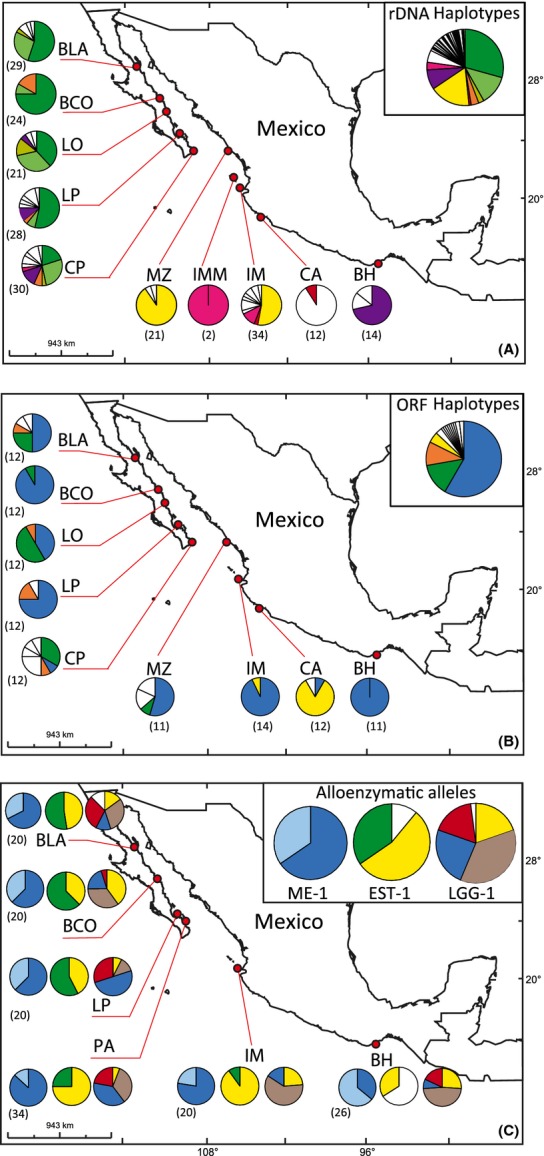
Sampling localities (red circles) and haplotype and allele frequencies from (A) rDNA, (B) a nuclear ORF, and (C) three representative alloenzymatic loci (of five analyzed). Shared haplotypes or alleles are color coded, the rest are private (*n* = sample size).

The three types of genetic marker analyzed in this study showed contrasting levels of polymorphisms, in which the rDNA sequences were the most diverse and the allozymes the least. The nuclear rDNA sequences (*n* = 215 seqs. 555 bp) contained 18 variable sites, 13 parsimony-informative sites, and eight indels. A total of 35 haplotypes were found, only eight of which were shared among some localities (Table S4). Baja California locations shared five haplotypes and only two were shared with continental locations. Localities along the coast of southwestern Mexico shared three haplotypes and possessed fewer unique haplotypes compared to those in the peninsula (Fig. 3). The two colonies sampled from Isla María Madre shared a haplotype also found in Islas Marietas. Due to the limited sample size, this location was not included in further analyses.

The ORF sequences (*n* = 108; 363 bp) contained seven segregating sites, six of them parsimony informative. Fourteen ORF haplotypes were found in total, four of which were shared among some localities. The most widespread and prevalent occurred in 58% of the individuals (Table S5). Two haplotypes were shared between regions and one was characteristic to each region (ORF-2 green and ORF-6 yellow, Fig. 3). In contrast, two allozyme loci (*ME*-1 and *GDH*-1) were di-allelic, two (*GDH*-2 and *EST*-1) were tri-allelic, and one (*LGG*-1) was penta-allelic (Figs S5 and S6) in the 139 additional organisms genotyped using protein electrophoresis. All but two alleles were shared among localities; however, some revealed significant frequency shifts among regions (e.g., relative abundance of yellow *EST*-1 allele, Fig. 3; gray and blue *ME*-1 and *GDH*-1 alleles inside and outside of the Gulf, Fig. S5) and two of them were private (white *EST*-1 predominant in BH and white *LGG*-1 in BLA, Fig. 3). The patterns of genotype distribution paralleled that of the alleles with the presence of frequency shifts among regions and private genotypes (Fig. S6). These results reveal that, in consistence with rel-ACH predictions, central localities in Baja California have a similar genetic architecture, whereas peripheral continental corals are more heterogeneous among themselves and distinct from the abundant northern populations.

The geographic patterns of diversity among genetic markers were largely congruent but not identical, and revealed a trend of higher diversity levels in peninsular abundant coral populations. Even though mean levels of genetic diversity in continental localities did not significantly differ from those in the peninsula (rDNA *t*_162(0.05)_ = 0.51, *P* = 0.61; ORF *t*_95(0.05)_ = 1.40, *P* = 0.16; allozyme *t*_102(0.05)_ = 0.52, *P* = 0.60), the most diverse localities were located in Baja California (rDNA: BLA, LO, LP, and CP *h*_(12)_ ≥ 0.63; ORF: BLA, LO, and CP *h* ≥ 0.62; allozymes: BLA, BC, and LP *He* > 0.553; Table 2) and a larger fraction of the most diverse localities per genetic marker were found there (five of six in rDNA with *h*_(12)_ ≥ 0.63, three of four in ORF with *h* ≥ 0.62, and three of four in allozymes with *He* ≥ 0.553). Nucleotide diversity behaved similarly to haplotype diversity. Similar trends to *h* were found in haplotype richness of rDNA as LO, LP, and CP (*A*_(12)_ = 4.65, 5.48 and 6.94) were the most diverse in Baja California, and IM (*A*_(12)_ = 5.52) in the continent. Likewise, the highest haplotype richness of ORF were found in peninsular localities (BLA = 5 and CP = 7). In contrast, the total number of alloenzymatic alleles for all loci varied very little among localities (14 ≥ *A* ≥ 12) (Table 2).

To test diversity patterns according to rel-ACH predictions, we correlated the percent live coral cover with levels of genetic diversity (*h* and *A* for rDNA and ORF and *He* and *A* for allozymes). Even though the trends suggest a positive relationship of DNA sequence diversity with coral abundance (Fig. S7A–D), consistent with rel-ACH predictions, the only significant correlation was found in rDNA diversity as measured by *A*_(12)_ (*r* = 0.76, *P* = 0.02) (Fig. S7B). Mean levels of diversity of the five allozyme loci did not reveal any relationship with live coral cover (Fig. S7E–F). Careful analyses of the scatter plots revealed that the inconsistencies with rel-ACH were attributable to two samples. Carrizales (CA) had high coral cover but low diversity in rDNA and Mazatlan (MZ) had a very low coral cover but relatively high genetic diversity in ORF (Table 2; Fig. S7A–D). Notably, these outlying observations are responsible for the lack of significance in the above correlations (exclusion of CA yields for *h*_rDNA_ *r* = 0.84 *P* = 0.009 and increases *A*_rDNA_ *r* = 0.88 *P* = 0.004; exclusion of MZ yields for *h*_ORF_ *r* = 0.75 *P* = 0.03 and *A*_ORF_ *r* = 0.80 *P* = 0.02).

### Geographic patterns of genetic structure and gene flow

*Porites panamensis* exhibited significant population structure across the Mexican Pacific (Table 3A). Significant heterogeneity was observed among populations in all molecular markers and the degree of structure correlated with their level of polymorphism (rDNA *Φ*_st_ = 0.726 > ORF *Φ*_st_ = 0.385 > allozymes *F*_st_ = 0.138, all *P* < 0.01). Significant structure was also found between peninsular and continental regions in rDNA (*Φ*_ct_ = 0.437 *P* < 0.001) and allozymes (*F*_ct_ = 0.050 *P* = 0.02), ORF being marginally nonsignificant (*Φ*_ct_ = 0.116 *P* = 0.058); and finally among populations within regions (rDNA *Φ*_sc_ = 0.512; ORF *Φ*_sc_ = 0.304; allozymes *F*_sc_ = 0.093, all *P* < 0.001). Differentiation among localities in each region revealed a higher degree of genetic structuring among continental (rDNA *Φ*_st_ = 0.795; ORF *Φ*_st_ = 0.550; allozymes *F*_st_ = 0.181, all *P* < 0.001) than among peninsular (rDNA *Φ*_st_ = 0.136; allozymes *F*_st_ = 0.062, both *P* < 0.001; and ORF *Φ*_st_ = 0.169 *P* < 0.01) populations (Table 3B, C). Pairwise *Φ*_st_ corroborated the existence of significantly high levels of genetic differentiation between regions and among populations within regions (Table 4). Almost all of the pairwise *Φ*_st_ involving continental samples were significant; whereas this only occurred in half of the comparisons between peninsular populations (rDNA: five of 10, ORF: three of 10, allozymes: three of six, which involved the southernmost localities of CP and PA Table 4).

**Table 3:** Hierarchical analysis of molecular variance (AMOVA) to estimate levels of genetic differentiation between regions (*Φ*_ct_), among localities within regions (*Φ*_sc_), and among all localities (*Φ*_st_) using DNA sequences, and analogous hierarchical analysis of variance (ANOVA, *F*-statistics) using allozymes

	rDNA (ITS)		ORF		Allozymes	
			
Source of variation	Variance components	% of variation	Variance components	% of variation	Variance components	% of variation
(A) Partitioning of genetic variation between regions						
Between regions	0.00111	43.73	0.00019	11.62	0.07550	5.01
Among localities within regions	0.00073	28.83	0.00044	26.84	0.13306	8.83
Within localities	0.00070	27.43	0.00101	61.54	1.29716	86.14
*Φ*_ct_ =	0.437 (*P* < 0.001)		0.116 (*P* > 0.05)		*F*_ct_ = 0.050 (*P* < 0.05)	
*Φ*_sc_ =	0.512 (*P* < 0.001)		0.304 (*P* < 0.001)		*F*_sc_ = 0.093 (*P* < 0.001)	
*Φ*_st_ =	0.726 (*P* < 0.01)		0.385 (*P* < 0.001)		*F*_st_ = 0.138 (*P* < 0.001)	
(B) Partitioning of genetic variation among peninsular localities						
Among localities	0.00013	13.63	0.00028	16.92	0.08901	6.25
Within localities	0.00083	86.37	0.00139	83.08	1.33493	93.74
*Φ*_st_ =	0.136 (*P* < 0.001)		0.169 (*P* < 0.01)		*F*_st_ = 0.062 (*P* < 0.001)	
(C) Partitioning of genetic variation among continental localities						
Among localities	0.00184	79.54	0.00065	54.96	0.27001	18.13
Within localities	0.00047	20.46	0.00053	45.04	1.21869	81.86
*Φ*_st_ =	0.795 (*P* < 0.001)		0.550 (*P* < 0.001)		*F*_st_ = 0.181 (*P* < 0.001)	

**Table 4:** Pairwise *Φ*_st_ and *F*_st_ between populations of *Porites panamensis* obtained from rDNA, ORF and allozyme analyses and linear geographic distance between localities in kilometers used in IBD analyses

	Pairwise *Φ*_st_ rDNA	Pairwise *Φ*_st_ ORF	Pairwise *F*_st_ alloenzymes	Distance, km
BLA vs. BC	**0.138**	0.096	0.001	305
BLA vs. LO	0.029	0.017	–	409
BLA vs. LP	0.045	0.049	0.001	621
BLA vs. PA	–	–	**0.038**	695
BLA vs. CP	**0.142**	0.114	–	736
BLA vs. MZ	**0.815**	0.088	–	953
BLA vs. IM	**0.723**	**0.235**	**0.081**	1216
BLA vs. CA	**0.858**	**0.548**	–	1438
BLA vs. BH	**0.688**	0.223	**0.124**	2406
BC vs. LO	**0.304**	0.341	–	102
BC vs. LP	0.018	0	0.014	314
BC vs. PA	–	–	**0.072**	388
BC vs. CP	**0.289**	**0.331**	–	428
BC vs. MZ	**0.895**	0.130	–	660
BC vs. IM	**0.786**	0.005	**0.086**	912
BC vs. CA	**0.921**	**0.712**	–	1133
BC vs. BH	**0.782**	0	**0.129**	2119
LO vs. LP	0.102	**0.267**	–	217
LO vs. CP	0.049	0.052	–	327
LO vs. MZ	**0.845**	0.213	–	559
LO vs. IM	**0.721**	0.490	–	808
LO vs. CA	**0.888**	**0.646**	–	1028
LO vs. BH	**0.678**	**0.489**	–	2000
LP vs. PA	–	–	**0.070**	75
LP vs. CP	**0.156**	**0.276**	–	119
LP vs. MZ	**0.779**	0.087	–	410
LP vs. IM	**0.704**	0.055	**0.141**	616
LP vs. CA	**0.806**	**0.669**	–	823
LP vs. BH	**0.594**	0.053	**0.167**	1798
PA vs. IM	–	–	0.018	600
PA vs. BH	–	–	**0.159**	1720
CP vs. MZ	**0.665**	**0.203**	–	307
CP vs. IM	**0.596**	**0.440**	–	498
CP vs. CA	**0.682**	**0.566**	–	706
CP vs. BH	**0.360**	**0.422**	–	1675
MZ vs. IM	0.113	**0.232**	–	119
MZ vs. CA	**0.969**	**0.573**	–	528
MZ vs. BH	**0.913**	0.224	–	1495
IM vs. CA	**0.809**	**0.753**	–	244
IM vs. BH	**0.763**	0	**0.154**	1215
CA vs. BH	**0.897**	**0.826**	–	978

Bold values are significant (*P* < 0.05) after sequential Bonferroni correction and (–) no data.

**Figure 4:**
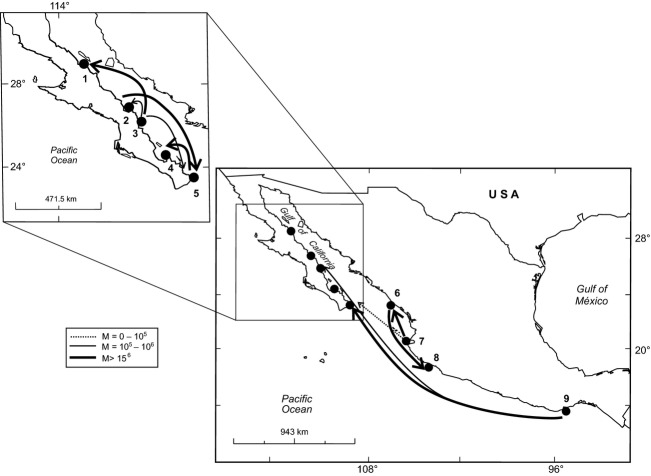
Direction and magnitude of gene flow between population pairs (black circles) of *Porites panamensis* estimated from rDNA sequences. Arrows represent directional gene flow and their relative magnitude is coded by line thickness. 1- BLA, 2- BC, 3- LO, 4- LP, 5- CP, 6- MZ, 7- IM, 8- CA, and 9- BH.

To assess gene flow patterns, we used a coalescence approach to estimate *M*. Attempts to estimate the full 81 parameter model with ORF sequence data were unsuccessful. Multiple MCMC runs yielded widely variable and inconsistent results pointing to a lack of convergence of the sampler. Presumably, the ORF data lacked sufficient variation for the estimation of all model parameters. Consequently, the following results were estimated with rDNA sequences. The highest levels of inferred gene flow were found among peninsular populations. In general, high gene flow occurred within regions and moderate levels of gene flow between regions (Fig. 4). The high gene flow estimated from continental BH to peninsular CP and LO stands in sharp contrast to the high levels of differentiation between regions. The high levels of estimated gene flow are presumably due to the presence of shared haplotype rDNA-10 dominant in BH (purple in Fig. 3; Table S3). This haplotype shows a cline of decreasing abundance toward the north in localities where it is present. Notably, there is a 1800 km gap between BH, where it dominates, and the peninsular localities (CP, LP, LO), where it becomes increasingly rare. Most gene flow values between localities were less than one or zero (Table S6). Three of nine simulated models were consistent among them in the general patterns and magnitude of gene flow, of which we present only one. The other six models were inconsistent in the estimated magnitudes but recovered the general patterns predicted by the rel-ACH. Gene flow patterns for allozymes, derived from *F*_st_ values under Wright's island model (*N*_*e*_*m* = (1/*F*_st_–1)/4) (Wright 1969), revealed that gene flow was moderate to high among peninsular populations and low among continental populations. Also, levels of gene flow between peninsular and continental samples were limited (Paz-García et al. 2008a).

### Environmental variables related to genetic patterns

**Figure 5:**
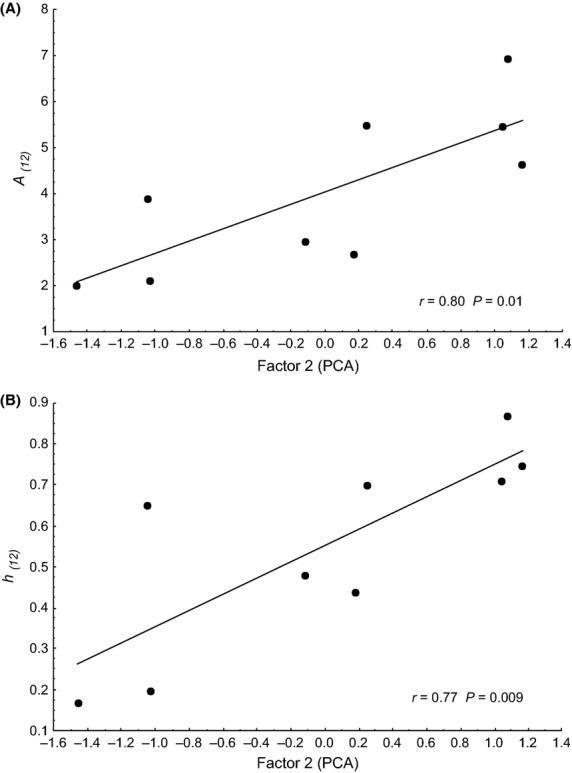
Correlation between genetic diversity of *Porites panamensis* using rDNA and Factor 2 of PCA. (A) Standardized haplotype richness (*A*_(12)_) versus Factor 2 and (B) standardized haplotype diversity index (*h*_(12)_) versus Factor 2.

The PCA applied to the 19 environmental variables produced three PCs that captured most (85.92%) of the environmental variance (PC1 = 56.84%, PC2 = 20.36%, and PC3 = 8.72%). To test the influence of environmental characteristics on standardized genetic diversity, we correlated the three principal components with *A* and *h* of rDNA and ORF, and with *He* and *A* of allozymes. Of the three PCs, only PC2 significantly correlated with *A* and *h* of rDNA (Fig. 5) and none with measures of ORF and allozyme diversity. PC2 had major factor loadings (FL) on chlorophyll *a* concentration (FL = −0.84), PAR (FL = 0.80), and light attenuation coefficient (FL = −0.87).

**Figure 6:**
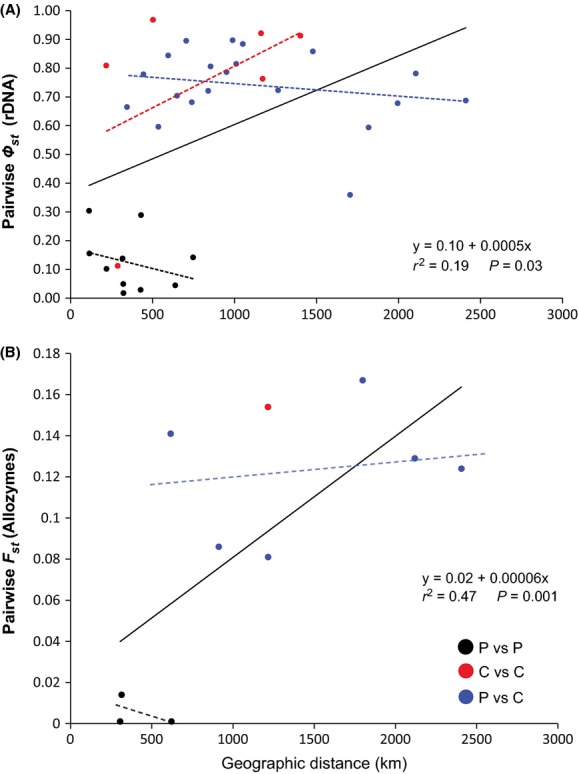
Isolation by distance of *Porites panamensis* in the Mexican Pacific. (A) *Φ*_st_ rDNA and (B) *F*_st_ allozyme is plotted as a function of geographic distance. P, peninsular localities, C, continental localities.

To test the influence of geographic and environmental distances on genetic patterns, we conducted partial Mantel tests among matrices of pairwise *Φ*_st_, geographic and environmental distances that revealed significant correlations between genetic differentiation and the geographical distance with rDNA (*P* = 0.03), but not between the former and environmental distance (Table 5). Moreover, the significant correlation between geographical and environmental distances (*P* = 0.02) revealed the presence of an environmental gradient. However, a detailed analysis of the scatter plot of *Φ*_st_ versus geographic distance shows that there is no significant correlation among peninsular (*r* = −0.28, *P* = 0.78, black points in Fig. 6A), among continental (*r* = 0.45, *P* = 0.27, red points in Fig. 6A), or between peninsular versus continental localities (*r*^*2*^ = 0.18, *P* = 0.49, blue points in Fig. 6A). Hence, the IBD signal over all data is driven by two clusters of comparisons showing markedly different patterns of genetic structure. On the one hand is a cluster of poorly differentiated peninsular populations (except for one continental comparison) and on the other a cluster of extremely differentiated populations encompassing both among-continental and peninsular-continental comparisons. We posit that this pattern reveals not stepping-stone dispersal consistent with IBD, but the influence of the GC acting as a major geographic barrier separating peninsular and continental populations. Partial Mantel test using allozyme data showed significant correlations between genetic differentiation and the geographical distance controlling for environmental Euclidean distance (*P* = 0.001; Table 5, Fig. 6B). These scatter plots of genetic versus geographic distance allozyme data are largely congruent with those obtained with rDNA (Fig. 6), hence they reflect the same processes described above. Remarkably, both patterns were obtained from independent loci and independent samples separated in time.

**Table 5:** Results of partial Mantel tests between matrices of genetic differentiation (*Φ*_st_ rDNA and *F*_st_ allozymes), linear geographic distance (km), and environmental distance (Euclidean distances)

	rDNA		Allozyme	
		
	*r*	*P* value	*r*	*P* value
1. Correlation of Φ_st_ and Euclidean distance	0.21	0.21	−0.07	0.53
2. Partial correlation of Φ_st_ and km, controlling for Euclidean distance matrix	0.40	0.07	0.84	0.001
3. Partial correlation of Φ_st_ and Euclidean distance matrix, controlling for km.	−0.05	0.51	−0.67	0.98
4. Correlation of Φ_st_ and km	0.44	0.03	0.68	0.059

## Discussion

The geographic patterns of abundance, genetic diversity, differentiation, and gene flow among populations of *P. panamensis* are partially consistent with the rel-ACH, in which northern peninsular populations represent the center of abundance and southern populations along the Mexican continental margin are peripheral. On the other hand, some expected patterns, such as isolation by distance along a latitudinal gradient, are equivocal. We argue that this partial concordance with the biogeographic model results from the combined effects of the biological features of the species, such as a limited dispersal potential, habitat discontinuity, and evolutionary processes.

### Patterns of abundance and environmental variation

*P. panamensis* showed maximum levels of abundance in northern localities along the eastern rocky coast of the peninsula of Baja California, which represents its center of abundance. Consequently, the continental populations along the western continental coast of Mexico represent peripheral populations relative to the center of abundance, following the rel-ACH model.

The ENM revealed that the highest probability of widespread occurrence of *P. panamensis* is found in the GC, largely along the peninsular coast and at the entrance of the Gulf. Even though the model only predicts probability of occurrence, not abundance, high probability of occurrence coincided with coral cover (i.e., abundance) in several regions, with notable exceptions. Two of the sampled continental localities stand out as less suitable for *P. panamensis*, as per the MaxEnt model predictions. One is Mazatlan, Sinaloa, representing the northernmost limit of distribution of the species on the Pacific continental margin. The coast of the state of Sinaloa is mostly sedimentary and is subject to strong fluctuations in sediment deposition and resuspension due to wind-wave action and considerable runoff. Near Mazatlan, these unstable and unfavorable conditions for corals are mainly responsible for their rarity. Coral forms are mainly incrusting (such as *Porites*) and coral reef structures are conspicuously absent. The second is Carrizales, Colima, which has the lowest probability of occurrence of all sampled locations yet has a coral cover comparable to the much more environmentally friendly Islas Marietas in Bahía de Banderas, Jalisco (Fig. 2). Carrizales is a small bay that may have limited connectivity with the rest of localities constituting a singular locality for hermatypic corals, and populations in this area may be self-recruiting, promoting inbreeding and loss of genetic diversity. In the case of *Pavona gigantea,* this locality was also strongly differentiated (Saavedra-Sotelo et al. 2011). A probability of occurrence hotspot off the coast of Guerrero (16° 48′ 22″ N, 99° 51′ 7″ W) was not sampled in this study, whereas in the southernmost sampled locality (BH) the high probability of occurrence contradicted the extremely low abundance of *P. panamensis* in an otherwise healthy and abundant coral reef system (Fig. 2). The low abundance of *P. panamensis* in these southern localities with suitable environmental conditions, as per the model predictions, suggests that biological and ecological factors, not included in the model, may be limiting the abundance of *P. panamensis* in the area (Elith et al. 2011). Interspecific competition may be a likely factor, as deduced from the increasing dominance of *P. panamensis* in the coral communities from the peripheral (i.e., southern) to the abundant central region to the north, where this species becomes increasingly dominant (Fig. 2).

Temperature, water clarity, and hard substrate availability show contrasting patterns in central and peripheral populations, which relate to the major variables determining the presence of *P. panamensis* according to the ENM. Abundance patterns of tropical hermatypic corals generally follow a latitudinal gradient, with higher abundances at low latitudes decreasing toward their distributional limits at higher latitudes (Hughes et al. 1999), as seen in our total coral cover data along the Mexican Pacific (black columns in Fig. 2). However, the pattern for *P. panamensis* is inverted. This is may be explained by the apparently singular thermal physiology of this species. Low temperature is a major limiting environmental variable for the development of corals (Harriott 1999; Kleypas et al. 1999). Even though there is no experimental evidence on the thermal tolerance of *P. panamensis*, its natural history suggests it is a more cold adapted than other stony corals in the ETP (Reyes-Bonilla 1993; Glynn and Ault 2000). The Mexican Pacific is characterized by seasonal latitudinal thermal gradients. In the GC, SST ranges from 17°C in the upper Gulf to 25°C at the mouth of the Gulf in winter; this gradient is lost in summer (Lavín and Marinone 2003; Lavín et al. 2003). In contrast, the tropical Mexican Pacific is characterized by a warm pool with an average SST of 28°C, reaching the mouth of the GC in September–October and retracting in April due to the increased intensity of California Current (Argote et al. 1995; Fiedler and Talley 2006). Accordingly, *P. panamensis* populations inhabiting the ETP have suffered significant mortalities during ENSO events. Subsequent to the 1992–1993 ENSO, the species nearly disappeared from some reefs in the Gulf of Chiriquí in Panamá and suffered moderate mortalities in the tropical Mexican Pacific (Glynn et al. 1994; Glynn 2000). After the 1997–1998 ENSO, the least affected coral communities in Mexico were in the GC, dominated by *P. panamensis*, where localized upwelling zones may have protected some populations from sea surface warming (Glynn 2000; Carriquiry et al. 2001; Reyes-Bonilla et al. 2002).

Water clarity is another essential feature for the maintenance of the symbiotic association between zooxanthellate endosymbionts and their cnidarian hosts. Water clarity decreases with the amount of particulate and dissolved matter in the water column. The Baja California peninsular coast is subject to semi-desertic weather conditions and features little or no input of particulate matter by riverine or pluvial runoff. In contrast, the continental coast is characterized by a tropical climate with marked rainy seasons and large expanses of coastal lagoons and freshwater runoff, bringing higher sediment and particulate organic matter loads to coastal waters decreasing water clarity (Gutiérrez-Estrada and Ortiz-Perez 2010). Different *Symbiodinium* clades differ in photosynthetically optimal irradiance; hence, differential use of light by symbionts is highly relevant for the diversification, abundance, and distribution of zooxanthellate corals (Iglesias-Prieto et al. 2004). In the GC, *P. panamensis* colonies have shown changes in composition of *Symbiodinium* clades with water depth (LaJeunesse et al. 2008; Paz-García et al. 2008b), however, the geographic pattern of variation of the endosymbiotic community of *P. panamensis* remains unknown. Finally, geographical differences in hard substrate availability, critical for coral settlement and recruitment, are contributing to the differentiation of central peninsular coasts, featuring mostly rocky shores that have allowed the success of the massive *P. panamensis*, and the peripheral continental coast featuring large expanses of soft sediments unsuitable as coral habitat (Fig. 2) (Riginos and Nachman 2001; Gutiérrez-Estrada and Ortiz-Perez 2010).

### rel-ACH predictions: genetic diversity patterns

The patterns of genetic diversity of *P. panamensis* partially agree with the predictions of the rel-ACH. Even though the three genetic markers show different levels of polymorphism, the patterns are largely congruent, which points to their validity as real and marker-independent features of sampled populations. High genetic diversity is found in regions with high coral abundance along the peninsular coast (Table 2), which is consistent with the large effective population size (*Ne*) expected in central populations. This holds true in the case of *P. panamensis*, as peninsular populations manifested both large model-based estimations of *θ* (mutation-scaled *Ne*) and high values of genetic diversity (Table S6). However, the high correlation between diversity and abundance breaks down with the inclusion of atypical localities such as MZ and CA, with the former bearing excess and the latter a deficit of diversity (Table 2; Fig. S7). These localities may reflect the presence of unfavorable conditions for the survival of *P. panamensis* along the continental coast (MZ) or high levels of inbreeding (CA), as discussed earlier.

The genetic diversity patterns predicted by the rel-ACH have been found in reefs from Australia and the Caribbean. Some corals species on the Great Barrier Reef have shown a markedly low genetic diversity in peripheral and isolated populations (Lord Howe Island) away from their center of abundance (Ayre and Hughes 2000, 2004; Miller and Ayre 2004; Noreen et al. 2009). Something similar was found in the Caribbean, northern populations of *Acropora palmata* tended to have lower levels of genotypic diversity mostly due to asexual reproduction (Baums et al. 2006). On large scale in the Atlantic Ocean, southern populations of *Montastraea cavernosa* showed low genetic diversity due a combined effect of small *Ne* and genetic drift or inbreeding depression (Nunes et al. 2009). In the ETP, the genetic patterns of hermatypic corals have only recently begun to be understood, however, there is evidence of decreased genetic diversity in peripheral populations of *Pavona gigantea* in the Mexican Pacific (Saavedra-Sotelo et al. 2011) and of *Porites lobata* in some localities of Costa Rica and Ecuador (Baums et al. 2012).

A significant association of environmental factors with levels of genetic diversity suggests an indirect effect through abundance and genetic drift, assuming neutral dynamics, or a direct adaptive effect, under selective conditions. In our study, genetic diversity using rDNA of the host correlates with environmental variables not only favoring the survival of the holobiont, such as temperature and substrate availability, but also impinging on water clarity: chlorophyll *a* concentration (negative covariation), PAR (positive covariation), and light attenuation coefficient (negative covariation). This suggests that increased water clarity favoring photosynthetic output by the symbionts is playing a role in favoring coral survivorship and reproduction leading to increased genetic diversification. Admittedly, determining the taxonomic/evolutionary identity and ecological attributes of *P. panamensis* endosymbionts across the Mexican Pacific is essential to understanding the mechanisms behind the patterns unveiled in the genetic architecture of the cnidarian host of this study. Collaborative efforts are currently being directed toward that goal.

### rel-ACH predictions: patterns of genetic structure and gene flow

Patterns of genetic subdivision within *P. panamensis* partially agree with predictions of the rel-ACH. Genetic differentiation was pervasive throughout the studied area and most patterns were consistent not only among the three molecular markers but also between two temporal samples (allozymes: 2004–2006; DNA: 2008–2011), suggesting that they are not marker dependent and stable in recent time. Genetic architecture among abundant peninsular populations contrasted with those at the periphery (Fig. 3; Table 3), both in terms of genetic differentiation between peninsular and continental populations (Table 3A) and patterns within each region (Fig. 4; Table 4). Similar results were found in populations of *Acropora palmata* and *Montastraea cavernosa*, which showed significant geographical subdivision due to the presence of peripheral populations with different genetic architecture (Baums et al. 2005, 2006; Nunes et al. 2009). Peninsular populations experienced high rates of gene flow, whereas genetic differentiation was mostly accounted for by one southern locality (CP, Fig. 4, Table 4). In contrast, most pairwise comparisons of continental populations were significant and consequently gene flow levels were very small (Table 4; Fig. 4). These results agree with reproduction studies in both regions; peninsular populations have several reproductive peaks throughout the year (Glynn et al. 1994; Mora-Pérez 2005); hence, the likelihood for long-distance dispersal is higher than in continental populations, where only one reproductive peak has been documented in summer (Carpizo-Ituarte et al. 2011; Rodríguez-Troncoso et al. 2011).

Our results suggest that continental populations are not only isolated from peninsular populations, but also among themselves. Although geographic distance seemed to be responsible for population divergence at a large spatial scale (Fig. 6), there was no evidence of IBD at smaller spatial scales. This suggests that genetic isolation results more from the differentiation of two geographic clusters (central and peripheral) separated by a significant habitat discontinuity (i.e., the GC acting as a dispersal barrier) than from the cumulative effects of geographic distance per se. Several phylogeographic studies across marine populations in the region suggest not only that the southern GC has acted as a barrier to gene flow and migration since the end of the Pleistocene, which is reflected in significant genetic divergence between populations in the Pacific coast of the Baja California peninsula and inside the Sea of Cortez (Terry et al. 2000; Stepien et al. 2001; Bernardi et al. 2003; Sandoval-Castillo et al. 2004; Muñiz-Salazar et al. 2005; Sandoval-Castillo and Rocha-Olivares 2011; Castillo-Páez et al. 2013), but also that ecological and evolutionary diversifying forces have acted on GC populations to promote genetic differentiation (Riginos and Nachman 2001; Riginos 2005; Segura et al. 2006). Thus, the biological and ecological factors leading to the observed patterns of genetic structure and gene flow can be summarized as follows. First, *P. panamensis* produces short-lived lecithotrophic planulae that settle near the parental colony, limiting the long-distance dispersal potential (Glynn et al. 1994). Second, continental populations are separated from peninsular populations by the GC, acting as a barrier for this poorly dispersing coral and promoting their genetic distinctiveness of the two regions (Fig. 3). Isolation is mediated by mesoscale oceanographic features inside the GC and at the entrance acting as barriers to dispersal of planktonic larvae. Circulation inside the gulf shifts seasonally from cyclonic to anticyclonic promoting the generation of mesoscale eddies entrapping larvae and favoring their retention (Brogan 1994; Peguero-Icaza et al. 2008; Contreras-Catala et al. 2012). At the entrance, convergence of surface water from the equator and the California Current flowing southward (Castro et al. 2000) promotes oceanic fronts blocking the dispersal of propagules. Third, within regions, the rocky shores of the western the GC provide a potentially continuous habitat for larval settlement within a permissive environment (Fig. 2), whereas barriers to dispersal such as stretches of unsuitable turbid habitat along the continental coast significantly limit connectivity. This results in higher isolation and genetic differentiation among continental than peninsular populations.

Although the pattern of genetic structure matches with the overall predictions of the rel-ACH, the high levels of inferred gene flow from BH to peninsular CP and LO is not only contradictory but also improbable, given the limited dispersal of this species. Reconciliation of this result requires considering the ghosts of dispersal past (Hellberg 1995; Benzie 1999). Given that our analytical approach involved coalescent modeling of gene trees it is sensitive to the influence of historical demography and connectivity patterns. Careful examination of haplotype geographic distribution reveals that this connectivity is mediated by haplotype rDNA-10 (purple Fig. 3, Table S4). rDNA-10 is the dominant and only shared haplotype present in BH; it reappears 1800 km away as a rare haplotype in the three southernmost localities of Baja California (CP, LP, and LO). This is consistent with a scenario in which a formerly widespread polymorphism has survived in only a few peripheral populations where it has drifted to a high frequency (in BH) and disappeared in the rest. In central populations, owing to their larger *Ne*, this polymorphism has endured at much lower frequencies. This hypothetical scenario is also supported by the locus *LGG*-1, in which the allele D present throughout the gulf, is absent from IM to be found again in BH (Fig. S5). Similar patterns of structure and connectivity have been observed in sympatric populations of *Pavona gigantea* (Saavedra-Sotelo et al. 2011).

Finally, the large heterozygote deficit found in allozyme loci warrants caution in the interpretation of the numerical *F*_st_ values. This could be the result of inbreeding, clonality or null alleles. However, we think that this does not interfere with their power to corroborate the genetic structure patterns observed in the other loci. Indeed, in the worst-case scenario of the presence of null alleles, it is extremely unlikely that their random effect across loci and populations would be responsible for the geographic patterns observed, which largely coincides with an independent sample and loci unaffected by this problem.

### Coda

We set out to test the predictions of the rel-ACH in a dominant hermatypic coral inhabiting the reef communities of the ETP. Our genetic seascape analyses, integrating niche modeling, environmental, and genetic patterns from three molecular markers with complementary evolutionary rates, allowed us to unveil that the genetic architecture of *P. panamensis* conforms to a large extent to the expectations of the relaxed biogeographical model. In addition, our results clearly revealed added complexity stemming from significant habitat discontinuities in the face of a limited dispersal and outlier localities, but also, and perhaps more importantly, they allowed us to identify the key habitat covariates, such as temperature and photosynthetically relevant variables, that are influencing the genetic patterns of diversification and structure, and may hold the key to the survival of the species in the face of widespread environmental degradation.

## Data Accessibility

DNA sequences: from GenBank (Accession: KC178837-71 & KC866302-15). Environmental covariates, coral cover data and input files to Migrate software: all have been deposited in Dryad (http://datadryad.org) (DOI: 10.5061/dryad.67jg4).

## Supporting Information

**Table S1.** Localities, sampling dates and total number (*n*) of 25 m-long transects conducted at each site.

**Table S2.** PCR conditions and thermal profiles (TP) for rDNA and ORF.

**Table S3.** List of colonies with intragenomic polymorphisms and results of haplotype reconstructions using PHASE, fastPHASE and HAPAR (see M&M).

**Table S4.** Distribution of 35 rDNA haplotypes of *Porites panamensis* in populations in the tropical Mexican Pacific.

**Table S5.** Distribution of 14 ORF haplotypes of *Porites panamensis* in populations in the tropical Mexican Pacific.

**Table S6.** Estimates of effective population size (*θ* = 2 *Ne µ*) and migration rates between populations (*M* = *m/µ*). The migration rates include 95% confidence intervals. The arrows indicate the direction of gene flow and data is marked with a rectangle representing the highest values.

**Figure S1.** Scatter plots and regression lines of percent live coral cover against surveying effort (number of transects). ANOVA of linear regressions: total live cover *F* = 18.29 *P* = 0.004, *Porites panamensis* live cover *F* = 0.009 *P* = 0.926.

**Figure S2.** Intragenomic polymorphism due to a four base-pair indel. Variation is readily detected by observation of double peaks in some traces. (A) trace representing haplotype 1, (B) trace representing haplotype 4 and (C) trace with double signal (haplotypes 1 and 4) showing polymorphic base calls (arrows point to the “secondary” sequence).

**Figure S3.** Single nucleotide polymorphic position showing a double peak. (A) sequence with only one base call, and (B) sequence with double peak.

**Figure S4.** Correlation between percentage of live coral cover of *Porites panamensis* and degree latitude.

**Figure S5.** Frequency of alloenzymatic alleles. Five enzymatic systems (*ME-1*, *GDH-1*, *GDH-2*, *EST-1* and *LGG-1*). Localities: Bahía de los Ángeles (BLA), Bahía Concepción (BC), Bahía de la Paz (LP), Punta Arenas (PA), Islas Marietas (IM) and Bahías de Huatulco (BH).

**Figure S6.** Frequency of alloenzymatic genotypes. Five enzymatic systems (*ME-1*, *GDH-1*, *GDH-2*, *EST-1* and *LGG-1*). Localities: Bahía de los Ángeles (BLA), Bahía Concepción (BC), Bahía de la Paz (LP), Punta Arenas (PA), Islas Marietas (IM) and Bahías de Huatulco (BH).

**Figure S7.** Correlations between genetic diversity (Haplotype diversity – *h* – and standardized *h*_(12)_ ; Haplotype or allelic richness – *A* – and standardized *A*_(12)_; and expected heterozygosity – *He* –) and percentage live coral cover of *Porites panamensis*. (A, B) rDNA, (C, D) ORF and (E, F) allozymes.
