# Supplementary material for: Testing the genetic predictions of a biogeographical model in a dominant endemic Eastern Pacific coral (Porites panamensis) using a genetic seascape approach
Source: Ecol Evol. 2013 Sep 20;3(12):4070–91. doi: 10.1002/ece3.734 (PMC3853554; doi:10.1002/ece3.734)
Supplement: Supplementary file 1 [file ece30003-4070-SD1.docx]

Electronic Supplementary material

**Testing the genetic predictions of a biogeographical model in a dominant endemic Eastern Pacific coral (*Porites panamensis*) using a genetic seascape approach**

*Nancy C. Saavedra-Sotelo, Luis E. Calderón-Aguilera, Héctor Reyes-Bonilla, David A. Paz-García, R. Andrés López-Pérez, Amilcar Cupul-Magaña, Jose A. Cruz-Barraza, and Axayácatl Rocha-Olivares*

**CONTENTS**

**Table S1**. Localities, sampling dates and total number (n) of 25m-long transects conducted at each site.

**Table S2.** PCR conditions and thermal profiles (TP) for rDNA and ORF.

**Table S3.** List of colonies with intragenomic polymorphisms and results of haplotype reconstructions using PHASE, fastPHASE and HAPAR (see M&M).

**Table S4.** Distribution of 35 rDNA haplotypes of *Porites panamensis* in populations in the tropical Mexican Pacific.

**Table S5.** Distribution of 14 ORF haplotypes of *Porites panamensis* in populations in the tropical Mexican Pacific.

**Table S6.** Estimates of effective population size (*θ =2 Ne µ*) and migration rates between populations (*M = m/µ*). The migration rates include 95% confidence intervals. The arrows indicate the direction of gene flow and data is marked with a rectangle representing the highest values.

**Figure S1.** Scatter plots and regression lines of percent live coral cover against surveying effort (number of transects). ANOVA of linear regressions: total live cover *F* = 18.29 p = 0.004, *Porites panamensis* live cover *F* = 0.009 p = 0.926.

**Figure S2**. Intragenomic polymorphism due to a four base-pair indel. Variation is readily detected by observation of double peaks in some traces. a) trace representing haplotype 1, b) trace representing haplotype 4 and c) trace with double signal (haplotypes 1 and 4) showing polymorphic base calls (arrows point to the “secondary” sequence).

**Figure S3**. Single nucleotide polymorphic position showing a double peak. a) sequence with only one base call, and b) sequence with double peak.

**Figure S4**. Correlation between percentage of live coral cover of *Porites panamensis* and degree latitude.

**Figure S5**. Frequency of alloenzymatic alleles. Five enzymatic systems (*ME-1*, *GDH-1*, *GDH-2*, *EST-1* and *LGG-1*). Localities: Bahía de los Angeles (BLA), Bahía Concepción (BC), Bahía de la Paz (LP), Punta Arenas (PA), Islas Marietas (IM) and Bahías de Huatulco (BH).

**Figure S6**. Frequency of alloenzymatic genotypes. Five enzymatic systems (*ME-1*, *GDH-1*, *GDH-2*, *EST-1* and *LGG-1*). Localities: Bahía de los Angeles (BLA), Bahía Concepción (BC), Bahía de la Paz (LP), Punta Arenas (PA), Islas Marietas (IM) and Bahías de Huatulco (BH).

**Figure S7**. Correlations between genetic diversity (Haplotype diversity – *h* – and standardized *h_(12)_* ; Haplotype or allelic richness – *A* – and standardized *A_(12)_*; and expected heterozygosity – *He* –) and percentage live coral cover of *Porites panamensis*. a-b) rDNA, c-d) ORF and e-f) allozymes.

**Table S1.** Localities, sampling dates (mo/20yr) for ecological surveys and tissue collections and total number of 25m-long transects conducted at each site (tr).

| **Locality** | **Code** | **Ecological** | **Tissue** | **tr** |
| --- | --- | --- | --- | --- |
| 1. Bahía de los Angeles | BLA | 11/10 | 02,03/09 | 3 |
| 2. Bahía Concepción | BC | 02/11 | 03/10 | 3 |
| 3. Loreto | LO | 11/10 | 11/11 | 3 |
| 4. La Paz | LP | 11/08 | 08/08 | 12 |
| 5. Cabo Pulmo | CP | 11/08, 10/10; 05/11 | 08/08 | 48 |
| 6. Mazatlán | MZ | 07/08 | 09/10 | 1 |
| 7. Islas Marietas | IM | 10,11/10, 03,05/11 | 06/10 | 72 |
| 8. Carrizales | CA | 02/10, 07/11 | 07/11 | 26 |
| 9. Bahías de Huatulco | BH | 11/09, 09/10 | 09/08 | 84 |

**Figure S1.** Scatter plots and regression lines of percent live coral cover against surveying effort (number of transects). ANOVA of linear regressions: total live cover *F* = 18.29 p = 0.004, *Porites panamensis* live cover *F* = 0.009 p = 0.926.

**Table S2.** PCR conditions and thermal profiles (TP) for rDNA and ORF.

| **PCR conditions:** |  | **TP (rDNA)** | | |  | **TP (ORF)** | | |  |
| --- | --- | --- | --- | --- | --- | --- | --- | --- | --- |
| - 0.18 mM dNTPs |  | *1.* | 94**°** | 1 min |  | *1.* | 94**°** | 2 min | *Initial heating* |
| - 1X PCR buffer (10 mM Tris HCl, 50 mM KCl and 1.5 mM MgCl_2_) |  | *2.* | 36 cycles of: |  |  | *2.* | 30 cycles of: |  |  |
| - 0.4 µM of each primer |  |  | 94**°** | 1 min |  |  | 94**°** | 45 sec | *Denaturation* |
| - 1 U *Taq* DNAPol (NEB, Ipswich, MA) |  |  | 55**°** | 1 min |  |  | 50**°** | 45 sec | *Annealing* |
| - 42 ng of template DNA |  |  | 72**°** | 2 min |  |  | 72**°** | 2 min | *Polymerization* |
| Total volume: 25 µl. |  | *3.* | 72**°** | 9 min |  | *3.* | 72**°** | 5 min | *Final elongation* |

Primers sequences: ITS-4 (TCCTCCGCTTATTGATATGC), ITS-5 (GGAAGTAAAAGTCGTAACAAGG), MM32f2 (GGTACTATCCCAAAGTTAGAGGTGA) and MM32r2 (GTTTTTATGGATGCGGTTCTTTAC). Quality and quantity of PCR products were assessed by electrophoresis in 1.5% agarose gels. Subsequently, PCR products were purified with QIAquick PCR Purification Kit (QIAGEN) and sequenced using PCR primers and Big Dye Terminator v.3.1 chemistry in an ABI 3730xl (Applied Biosystems). Base calling was verified with CodonCode Aligner® v3.7.1.1 (CodonCode Corp.).

Intragenomic rDNA polymorphisms were found in 33 colonies of *Porites panamensis* and were unequivocally resolved using careful inspection of chromatograms and dedicated algorithms (Table S2). Only two kinds or polymorphisms were detected: a four base-pair insertion (Figure S2) and SNPs (Figure S3).

**Figure S2**. Intragenomic polymorphism due to a four base-pair indel. Variation is readily detected by observation of double peaks in some traces. a) trace representing haplotype 1, b) trace representing haplotype 4 and c) trace with double signal (haplotypes 1 and 4) showing polymorphic base calls (arrows point to the “secondary” sequence).

**Figure S3**. Single nucleotide polymorphic position showing a double peak. a) Sequence with only one base call, and b) sequence with SNP.

**Table S3.** List of colonies with intragenomic polymorphisms and results of haplotype reconstructions using PHASE, fastPHASE and HAPAR (see M&M). The output probability of haplotype was 0.9.

| **Polymorphic colonies** | **Locality** | **Haplotype**  **reconstruction** | **Polymorphic positions** |
| --- | --- | --- | --- |
| PPABA-4 | Bahía de los Ángeles | Hap 1-2 | 141 |
| PPABA-11 | Bahía de los Ángeles | Hap 1-2 | 141 |
| PPABA-12 | Bahía de los Ángeles | Hap 1-2 | 141 |
| PPABA-56 | Bahía de los Ángeles | Hap 5-6 | 457 |
| PPALO-4 | Loreto | Hap 1-2 | 141 |
| PPALO-6 | Loreto | Hap 1-27 | (263-266)* |
| PPALO-17 | Loreto | Hap 4-28 | (455-458)* |
| PPALO-20 | Loreto | Hap 2-10 | 496 |
| PPALP-1 | La Paz | Hap 29-30 | 141 |
| PPALP-4 | La Paz | Hap 1-31 | (455-458)* |
| PPALP-7 | La Paz | Hap 10-32 | 141, 496, 512 |
| PPALP-8 | La Paz | Hap 10-33 | 106 |
| PPALP-10 | La Paz | Hap 10-32 | 141, 496, 512 |
| PPACP-2 | Cabo Pulmo | Hap 1-10 | 141, 496 |
| PPACP-3 | Cabo Pulmo | Hap 10-13 | 496, 497 |
| PPACP-4 | Cabo Pulmo | Hap 2-14 | 234 |
| PPACP-6 | Cabo Pulmo | Hap 1-2 | 141 |
| PPACP-9 | Cabo Pulmo | Hap 4-15 | 496 |
| PPACP-12 | Cabo Pulmo | Hap 2-14 | 234 |
| PPACP-17 | Cabo Pulmo | Hap 1-7 | 496 |
| PPACP-19 | Cabo Pulmo | Hap 2-10 | 496 |
| PPACP-21 | Cabo Pulmo | Hap 2-16 | 204 |
| PPAIM-5 | Islas Marietas | Hap 9-19 | 106, 234, (455-458)*, 496, |
| PPAIM-6 | Islas Marietas | Hap 19-20 | 454 |
| PPAIM-11 | Islas Marietas | Hap 22-23 | (455-458)* |
| PPAIM-17 | Islas Marietas | Hap 19-24 | 411 |
| PPAIM-21 | Islas Marietas | Hap 21-25 | 522 |
| PPAIM-24 | Islas Marietas | Hap 19-24 | 411 |
| PPAIM-28 | Islas Marietas | Hap 19-26 | 455 |
| PPAMZ-12 | Mazatlán | Hap 19-34 | 504 |
| PPAMZ-19 | Mazatlán | Hap 19-35 | 497 |
| PPAOX-3 | Bahías de Huatulco | Hap 11-12 | (263-266)* |
| PPAOX-7 | Bahías de Huatulco | Hap 11-12 | (263-266)* |

*Four base-pair indel


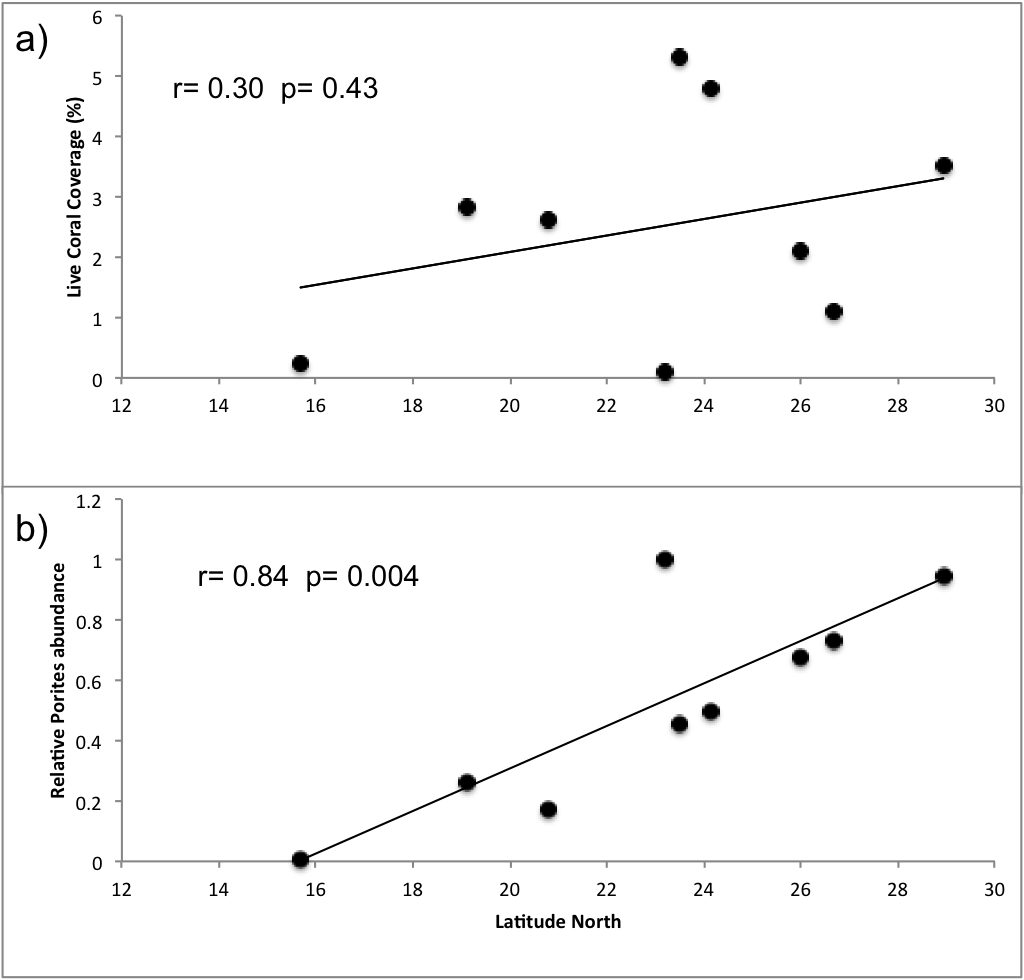


**Figure S4**. a) Correlation between percentage of live coral cover and latitude. b) Correlation between relative abundance and latitude.

**Table S4.** Distribution of 35 rDNA haplotypes of *Porites panamensis* in populations in the tropical Mexican Pacific.

| Haplotypes | Bahía de los Angeles | Bahía Concepción | Loreto | La Paz | Cabo Pulmo | Mazatlán | Islas Marietas | Islas Marías | Carrizales | Bahías de Huatulco | Total | GenBank accession |
| --- | --- | --- | --- | --- | --- | --- | --- | --- | --- | --- | --- | --- |
| rDNA-1 | 16 | 18 | 8 | 15 | 6 |  |  |  |  |  | 63 | KC178837 |
| rDNA-2 | 8 | 2 | 7 | 2 | 8 |  |  |  |  |  | 27 | KC178838 |
| rDNA-3 | 2 |  |  |  |  |  |  |  |  |  | 2 | KC178839 |
| rDNA-4 | 1 |  | 3 |  | 1 |  |  |  |  |  | 5 | KC178840 |
| rDNA-5 | 1 |  |  |  |  |  |  |  |  |  | 1 | KC178841 |
| rDNA-6 | 1 |  |  |  |  |  |  |  |  |  | 1 | KC178842 |
| rDNA-7 |  | 4 |  | 1 | 2 |  |  |  |  |  | 7 | KC178843 |
| rDNA-8 |  |  |  |  |  |  |  |  | 11 |  | 11 | KC178844 |
| rDNA-9 |  |  |  |  |  |  | 1 |  | 1 |  | 2 | KC178845 |
| rDNA-10 |  |  | 1 | 3 | 4 |  |  |  |  | 10 | 18 | KC178846 |
| rDNA-11 |  |  |  |  |  |  |  |  |  | 2 | 2 | KC178847 |
| rDNA-12 |  |  |  |  |  |  |  |  |  | 2 | 2 | KC178848 |
| rDNA-13 |  |  |  |  | 1 |  |  |  |  |  | 1 | KC178849 |
| rDNA-14 |  |  |  |  | 2 |  |  |  |  |  | 2 | KC178850 |
| rDNA-15 |  |  |  |  | 1 |  |  |  |  |  | 1 | KC178851 |
| rDNA-16 |  |  |  |  | 3 |  |  |  |  |  | 3 | KC178852 |
| rDNA-17 |  |  |  |  | 1 |  | 4 | 2 |  |  | 7 | KC178853 |
| rDNA-18 |  |  |  |  | 1 |  |  |  |  |  | 1 | KC178854 |
| rDNA-19 |  |  |  |  |  | 19 | 18 |  |  |  | 37 | KC178855 |
| rDNA-20 |  |  |  |  |  |  | 1 |  |  |  | 1 | KC178856 |
| rDNA-21 |  |  |  |  |  |  | 3 |  |  |  | 3 | KC178857 |
| rDNA-22 |  |  |  |  |  |  | 1 |  |  |  | 1 | KC178858 |
| rDNA-23 |  |  |  |  |  |  | 1 |  |  |  | 1 | KC178859 |
| rDNA-24 |  |  |  |  |  |  | 2 |  |  |  | 2 | KC178860 |
| rDNA-25 |  |  |  |  |  |  | 2 |  |  |  | 2 | KC178861 |
| rDNA-26 |  |  |  |  |  |  | 1 |  |  |  | 1 | KC178862 |
| rDNA-27 |  |  | 1 |  |  |  |  |  |  |  | 1 | KC178863 |
| rDNA-28 |  |  | 1 |  |  |  |  |  |  |  | 1 | KC178864 |
| rDNA-29 |  |  |  | 1 |  |  |  |  |  |  | 1 | KC178865 |
| rDNA-30 |  |  |  | 1 |  |  |  |  |  |  | 1 | KC178866 |
| rDNA-31 |  |  |  | 1 |  |  |  |  |  |  | 1 | KC178867 |
| rDNA-32 |  |  |  | 3 |  |  |  |  |  |  | 3 | KC178868 |
| rDNA-33 |  |  |  | 1 |  |  |  |  |  |  | 1 | KC178869 |
| rDNA-34 |  |  |  |  |  | 1 |  |  |  |  | 1 | KC178870 |
| rDNA-35 |  |  |  |  |  | 1 |  |  |  |  | 1 | KC178871 |
| Total | 29 | 24 | 21 | 28 | 30 | 21 | 34 | 2 | 12 | 14 | 215 |  |

**Table S5.** Distribution of 14 ORF haplotypes of *Porites panamensis* in populations in the tropical Mexican Pacific.

| Haplotypes | Bahía de los Angeles | Bahía Concepción | Loreto | La Paz | Cabo Pulmo | Mazatlán | Islas Marietas | Carrizales | Bahías de Huatulco | Total | GenBank accession |
| --- | --- | --- | --- | --- | --- | --- | --- | --- | --- | --- | --- |
| ORF-1 | 6 | 11 | 5 | 9 | 1 | 6 | 13 | 1 | 11 | 63 | KC866302 |
| ORF-2 | 1 |  | 1 | 2 | 1 |  |  |  |  | 5 | KC866303 |
| ORF-3 | 1 |  |  |  |  |  |  |  |  | 1 | KC866304 |
| ORF-4 | 3 | 1 | 6 |  | 4 | 1 |  |  |  | 15 | KC866305 |
| ORF-5 | 1 |  |  |  |  |  |  |  |  | 1 | KC866306 |
| ORF-6 |  |  |  |  |  |  | 1 | 10 |  | 11 | KC866307 |
| ORF-7 |  |  |  |  |  |  |  | 1 |  | 1 | KC866308 |
| ORF-8 |  |  |  |  | 3 |  |  |  |  | 3 | KC866309 |
| ORF-9 |  |  |  |  | 1 |  |  |  |  | 1 | KC866310 |
| ORF-10 |  |  |  |  | 1 |  |  |  |  | 1 | KC866311 |
| ORF-11 |  |  |  |  | 1 |  |  |  |  | 1 | KC866312 |
| ORF-12 |  |  |  | 1 |  |  |  |  |  | 1 | KC866313 |
| ORF-13 |  |  |  |  |  | 2 |  |  |  | 2 | KC866314 |
| ORF-14 |  |  |  |  |  | 2 |  |  |  | 2 | KC866315 |
| Total | 12 | 12 | 12 | 12 | 12 | 11 | 14 | 12 | 11 | 108 |  |


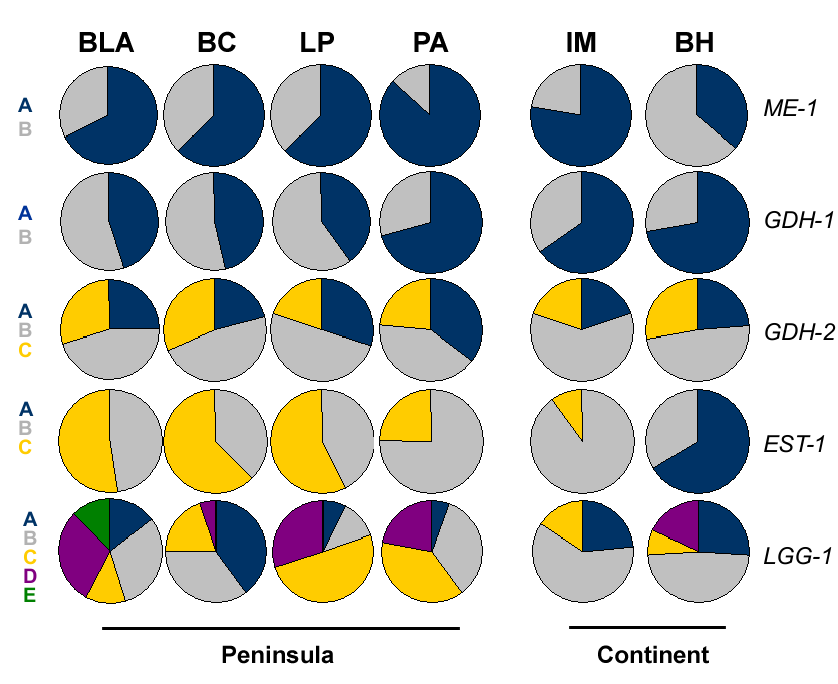


**Figure S5**. Frequency of alloenzymatic alleles. Five enzymatic systems (*ME-1*, *GDH-1*, *GDH-2*, *EST-1* and *LGG-1*). Localities: Bahía de los Angeles (BLA), Bahía Concepción (BC), Bahía de la Paz (LP), Punta Arenas (PA), Islas Marietas (IM) and Bahías de Huatulco (BH).


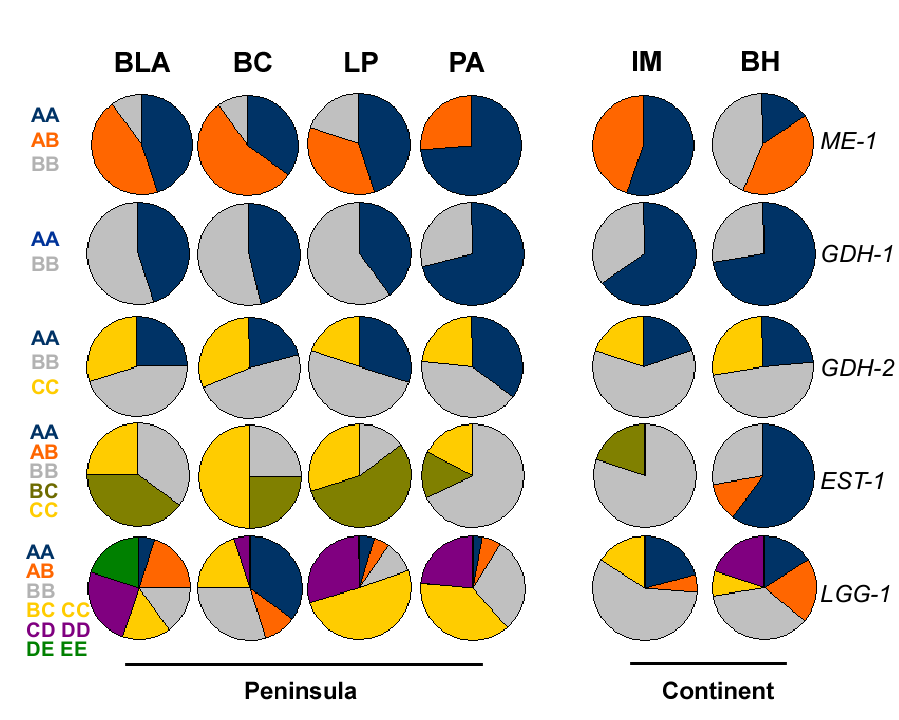


**Figure S6**. Frequency of alloenzymatic genotypes. Five enzymatic systems (*ME-1*, *GDH-1*, *GDH-2*, *EST-1* and *LGG-1*). Localities: Bahía de los Angeles (BLA), Bahía Concepción (BC), Bahía de la Paz (LP), Punta Arenas (PA), Islas Marietas (IM) and Bahías de Huatulco (BH).

**Figure S7**. Correlations between genetic diversity (Haplotype diversity – *h* – and standardized *h_(12)_* ; Haplotype or allelic richness – *A* – and standardized *A_(12)_*; and expected heterozygosity – *He* –) and percentage live coral cover of *Porites panamensis*. a-b) rDNA, c-d) ORF and e-f) allozymes.

**Table S6.** Estimates of effective population size (*θ =2 Ne µ*) and migration rates between populations (*M = m/µ*). The migration rates include 95% confidence intervals. The arrows indicate the direction of gene flow and data in bold representing the highest values.

|  | **n** | ***θ*** | ***M = m/ µ*** | | | | | | | | |
| --- | --- | --- | --- | --- | --- | --- | --- | --- | --- | --- | --- |
|  |  |  | **1→ *i*** | **2→ *i*** | **3→ *i*** | **4→ *i*** | **5→ *i*** | **6→ *i*** | **7→ *i*** | **8→ *i*** | **9→ *i*** |
| 1. Bahía de los Ángeles | 29 | **3.4 x 10^-3^**  (2.0 x10^-3^ – 6.2 x 10^-3^) | 0 | 6.7 x 10^-11^  (5.9 x 10^-11^ - 1.8 x 10^+5^) | **3,680,000**  (2.5 x 10^+6^ – 5.2 x 10^+6^) | 1.4 x 10^-10^  (1.2 x 10^-10^ – 3.7 x 10^+5^) | 1.6 x 10^-10^  (1.4 x 10^-10^ – 1.2 x 10^+5^) | 2.11 x 10^-10^  (1.8 x 10^-10^ – 1.2 x 10^+5^) | 2.7 x 10^-10^  (2.3 x 10^-10^ – 1.2 x 10^+5^) | 3.2 x 10^-10^  (2.8 x 10^-10^ – 1.2 x 10^+5^) | 5.3 x 10^-10^  (4.6 x 10^-10^ -1.2 x 10^+5^) |
| 2. Bahía Concepción | 24 | 2.6 x 10^-4^  (1.9 x 10^-4^ – 3.5 x 10^-4^) | 1.6 x 10^-9^  (1.4 x 10^-9^ – 2.4 x 10^+5^) | 0 | **892,439**  (5.6 x 10^+5^ – 1.3 x 10^+6^) | 1.7 x 10^-9^  (1.5 x 10^-9^ – 2.5 x 10^+5^) | 2.3 x 10^-9^  (2.0 x 10^-9^ – 3.4 x 10^+5^) | 3.5 x 10^-9^  (3.1 x 10^-9^ – 1.2 x 10^+5^) | 4.9 x 10^-9^  (4.3 x 10^-9^ – 1.2 x 10^+5^) | 6.1 x 10^-9^  (5.3 x 10^-9^ – 1.2 x 10^+5^) | 1.1 x 10^-8^  (1.0 x 10^-8^ – 1.2 x 10^+5^) |
| 3. Loreto | 21 | 5.2 x 10^-4^  (4.2 x 10^-4^ – 6.5 x 10^-4^) | 3.2 x 10^-8^  (2.9 x 10^-8^ – 9.8 x 10^+4^) | 8.0 x 10^-9^  (7.0 x 10^-9^ – 2.4 x 10^+4^) | 0 | 1.7 x 10^-8^  (1.5 x 10^-8^ – 5.2 x 10^+4^) | 2.6 x 10^-8^  (2.3 x 10^-8^ – 7.8 x 10^+4^) | 4.4 x 10^-8^  (3.9 x 10^-8^ – 1.3 x 10^+5^) | 6.4 x 10^-8^  (5.6 x 10^-8^ – 1.9 x 10^+5^) | 8.1 x 10^-8^  (7.1 x 10^-8^ – 2.5 x 10^+5^) | **707,183**  (1.7 x 10^+5^ – 1.9 x 10^+6^) |
| 4. La Paz | 28 | 5.0 x 10^-4^  (3.3 x 10^-4^ – 8.05 x 10^-4^) | 1.76 x 10^-9^  (1.5 x 10^-9^ – 1.2 x 10^+5^) | 8.9 x 10^-10^  (7.8 x 10^-10^ – 1.2 x 10^+5^) | 6.2 x 10^-10^  (5.4 x 10^-10^ – 1.2 x 10^+5^) | 0 | **3,040,000**  (1.9 x 10^+6^ – 4.6 x 10^+6^) | 1.2 x 10^-9^  (1.0 x 10^-9^ – 1.2 x 10^+5^) | 1.7 x 10^-9^  (1.5 x 10^-9^ – 1.2 x 10+5) | 2.3 x 10^-9^  (2.0 x 10^-9^ – 1.2 x 10+5) | 5.1 x 10^-9^  (4.5 x 10^-9^ – 1.2 x 10^+5^) |
| 5. Cabo Pulmo | 30 | **3.0 x 10^-2^**  (1.6 x 10^-2^ – 5.4) | 1.1 x 10^-7^  (1.0 x 10^-7^ – 1.2 x 10^+5^) | **1,160,000**  (8.1 x 10^+5^ – 1.6 x 10^+6^) | **314,776**  (1.6 x 10^+5^ – 5.3 x 10^+5^) | 1.8 x 10^-8^  (1.6 x 10^-8^ – 1.9 x 10^+4^) | 0 | 4.7 x 10^-8^  (4.2 x 10^-8^ – 4.9 x 10^+4^) | **59,142**  (5.2 x 10^+4^ – 2.1 x 10^+5^) | 1.1 x 10^-7^  (9.6 x 10^-8^ – 1.1 x 10^+5^) | **1,190,000**  (5.6 x 10^+5^ – 2.2 x 10^+6^) |
| 6. Mazatlán | 21 | 2.7 x 10^-4^  (1.8 x 10^-4^ – 4.5 x 10^-4^) | 2.2 x 10^-10^  (1.9 x 10^-10^ – 1.2 x 10^+5^) | 1.5 x 10^-10^  (1.3 x 10^-10^ – 1.2 x 10^+5^) | 1.3 x 10^-10^  (1.1 x 10^-10^ – 1.2 x 10^+5^) | 9.4 x 10^-11^  (8.2 x 10^-11^ – 1.2 x 10^+5^) | 7.0 x 10^-11^  (6.2 x 10^-11^ – 1.2 x 10^+5^) | 0 | **1,230,000**  (7.0 x 10^+5^ – 2.0 x 10^+6^) | 1.2 x 10^-10^  (1.1 x 10^-10^ – 1.2 x 10^+5^) | 3.4 x 10^-10^  (3.0 x 10^-10^ – 1.2 x 10^+5^) |
| 7. Islas Marietas | 34 | **2.4 x 10^-3^**  (1.9 x 10^-3^ – 3.1 x 10^-3^) | 3.8 x 10^-10^  (3.3 x 10^-10^ – 9.4 x 10^+4^) | 2.9 x 10^-10^  (2.5 x 10^-10^ – 7.1 x 10^+4^) | 2.5 x 10^-10^  (2.2 x 10^-10^ – 6.3 x 10^+4^) | 1.9 x 10^-10^  (1.7 x 10^-10^ – 4.8 x 10^+4^) | 1.6 x 10^-10^  (1.4 x 10^-10^ -3.9 x 10^+4^) | 3.7 x 10^-11^  (3.3 x 10^-11^ – 9.2 x 10^+4^) | 0 | 7.7 x 10^-11^  (6.7 x 10^-11^ – 1.9 x 10^+4^) | 3.8 x 10^-10^  (3.3 x 10^-10^ – 9.4 x 10^+4^) |
| 8. Carrizales | 11 | - 1. x 10^-5^   (7.0 x 10^-6^ – 1.8 x 10^-5^) | 1.1 x 10^-13^  (9.5 x 10^-13^ – 1.2 x 10^+5^) | 1.0 x 10^-13^  (9.2 x 10^-13^ – 1.2 x 10^+5^) | 1.0 x 10^-13^  (9.1 x 10^-13^ - 1.2 x 10^+5^) | 1.0 x 10^-13^  (9.0 x 10^-13^ - 1.2 x 10^+5^) | 9.9 x 10-14  (8.8 x 10^-14^ – 1.2 x 10^+5^) | **1,230,000**  (3.2 x 10^+5^ – 3.6 x 10^+6^) | 9.4 x 10^-14^  (8.4 x 10^-14^ – 1.2 x 10^+5^) | 0 | 1.0 x 10^-13^  (9.1 x 10^-13^ – 1.2 x 10^+5^) |
| 9. Bahías de Huatulco | 15 | 3.9 x 10^-4^  (2.8 x 10^-4^ – 5.8 x 10^-4^) | 2.2 x 10^-8^  (1.9 x 10^-8^ – 1.2 x 10^+5^) | 2.0 x 10^-8^  (1.7 x 10^-8^ – 1.2 x 10^+5^) | 1.9 x 10^-8^  (1.6 x 10^-8^ – 1.2 x 10^+5^) | 1.7 x 10^-8^  (1.5 x 10^-8^ – 1.2 x 10^+5^) | 1.5 x 10^-8^  (1.4 x 10^-8^ – 1.2 x 10^+5^) | 1.4 x 10^-8^  (1.2 x 10^-8^ – 1.2 x 10^+5^) | 1.1 x 10^-8^  (9.9 x 10^-8^ – 3.7 x 10^+6^) | 1.3 x 10^-9^  (1.1 x 10^-9^ – 2.7 x 10^+5^) | 0 |
